# Supercoiling-mediated feedback rapidly couples and tunes transcription

**DOI:** 10.1016/j.celrep.2022.111492

**Published:** 2022-10-18

**Authors:** Christopher P. Johnstone, Kate E. Galloway

**Affiliations:** 1 Department of Chemical Engineering, MIT, 25 Ames St., Cambridge, MA 02139, USA; 2 Lead contact

## Abstract

Transcription induces a wave of DNA supercoiling, altering the binding affinity of RNA polymerases and reshaping the biochemical landscape of gene regulation. As supercoiling rapidly diffuses, transcription dynamically reshapes the regulation of proximal genes, forming a complex feedback loop. However, a theoretical framework is needed to integrate biophysical regulation with biochemical transcriptional regulation. To investigate the role of supercoiling-mediated feedback within multi-gene systems, we model transcriptional regulation under the influence of supercoiling-mediated polymerase dynamics, allowing us to identify patterns of expression that result from physical inter-gene coupling. We find that gene syntax—the relative ordering and orientation of genes—defines the expression profiles, variance, burst dynamics, and inter-gene correlation of two-gene systems. Furthermore, supercoiling can enhance or weaken biochemical regulation. Our results suggest that supercoiling couples behavior between neighboring genes, providing a regulatory mechanism that tunes transcriptional variance in engineered gene networks and explains the behavior of co-localized native circuits.

## In brief

Supercoiling-mediated feedback couples the transcription of proximal genes. Here, Johnstone and Galloway provide a framework for integrating biochemical gene regulation with the biophysical effects of DNA supercoiling. This unified model provides design principles for improving the performance of gene networks, developing novel regulatory functions, and accessing previously inaccessible regulatory dynamics.

## Graphical Abstract

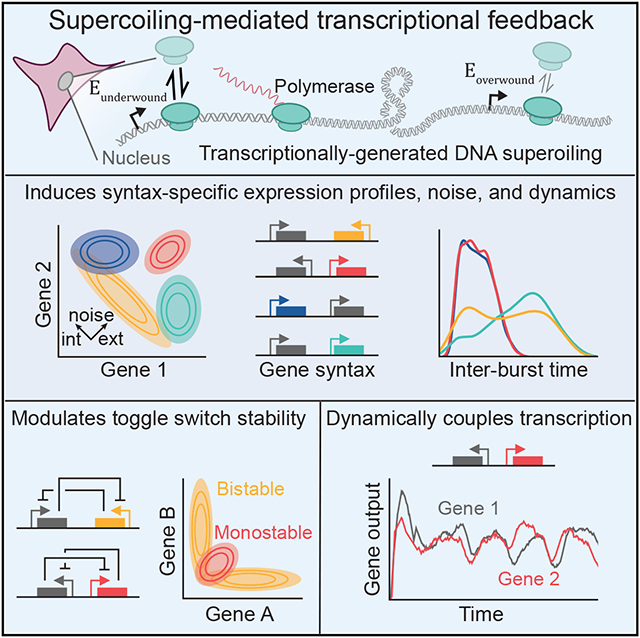

## INTRODUCTION

Cells coordinate complex behaviors through precise spatiotemporal control of gene expression. To rapidly advance gene- and cell-based therapies, synthetic biology aims to harness the power of native biology by constructing synthetic gene regulatory networks capable of dynamically prescribing cellular processes, states, and identities (Chen et al., 2012; Beitz et al., 2022; Purnick and Weiss, 2009; Elowitz and Lim, 2010). Synthetic networks process diverse inputs into complex logical and temporal responses (Weinberg et al., 2017; Xie et al., 2011; Tabor et al., 2009). From oscillators to pulse generators, synthetic circuits can precisely coordinate dynamic patterns of gene expression across populations of cells to control cell fate (Gardner et al., 2000; Elowitz and Leibler, 2000; Stricker et al., 2008; Danino et al., 2010; Ma et al., 2022; Park et al., 2019; Bashor et al., 2008; Galloway et al., 2013). However, rational *de novo* design of synthetic circuits remains challenging. Despite extensive bio-molecular modeling, integration of single genetic elements into systems often leads to emergent behaviors, requiring iterative design-build-test cycles to achieve the desired performance (Jones et al., 2020; Frei et al., 2020; Qian et al., 2017). Compounding the challenge, transcription exhibits significant extrinsic and intrinsic noise (To and Maheshri, 2010; Zopf et al., 2013; Desai et al., 2021). In particular, the stochastic nature of transcription makes coordinating expression across multiple genetic elements challenging (Rodriguez and Ren, 2019; Rodriguez and Larson, 2020; Quarton et al., 2020). Spatial variation in the nucleus and biochemical dynamics in condensates may contribute to bursting but provide limited parameters for tuning transcriptional noise (Henninger et al., 2020; Guo and Yang, 2019). Alternatively, mechanical sources of gene regulation offer one potential mechanism by which to understand and harness transcriptional noise to improve the predictable design of gene circuits (Johnstone and Galloway, 2021; Ancona et al., 2019; Kim et al., 2019; El Houdaigui et al., 2019; Meyer and Beslon, 2014).

The mechanical forces of DNA supercoiling powerfully shape transcriptional variance (Desai et al., 2021; Chong et al., 2014). In the process of transcription, RNA polymerases induce a leading wave of positive DNA supercoiling (Wu et al., 1988; Liu and Wang, 1987), reshaping the local structure of chromatin (Achar et al., 2020; Teves and Henikoff, 2014; Naughton et al., 2013; Guo et al., 2021). At the kilobase scale, chromatin structure correlates with gene regulation (HsiehTsung-Han et al., 2020; RowleyNichols et al., 2017; Krietenstein et al., 2020). In yeast and human cells, transcription-induced supercoiling demarks bounds of gene activity (Achar and Jagadheesh, 2020; Naughton et al., 2013; Kouzine et al., 2013). In particular, transcriptional activity dictates the strength of contact domains, indicating a role for transcription in forming and maintaining interactions at the kilobase scale (Rowley and Corces, 2018; RowleyNichols et al., 2017). Together these data suggest that the process of transcription drives formation of supercoiling-linked, kilobase-scale structures that feed back into transcriptional regulation of gene expression. As supercoiling rapidly diffuses across long distances (Loenhout and Dekker, 2012), transcriptional activity at one site may affect the overall activity and dynamics of transcription of proximal genes (Sevier and Levine, 2018; Sevier and Hormoz, 2022; Tripathi et al., 2021). Understanding how supercoiling induces coupling between neighboring genes provides the opportunity to improve the predictable design of transgenic systems from simple reporters to sophisticated dynamic circuits.

Here we develop a model of transcriptional regulation that integrates DNA supercoiling to examine how the orientation and placement of neighboring genes affects expression. Extending from a model of supercoiling-dependent polymerase motion (Sevier and Levine, 2018), our model includes the effects of supercoiling on polymerase binding and initiation. Specifically, we model RNA polymerase binding and initiation as a function of DNA supercoiling, such that underwound DNA favors RNA polymerase binding and overwound DNA limits binding. To extract experimentally testable predictions, we apply our model to simple two-gene systems that include a constitutive reporter and an inducible gene. Using these two-gene systems, we find that DNA supercoiling strongly influences the profile of gene expression and that influence is defined by syntax—the relative orientation and position of genetic elements—and the enclosing boundary conditions. In addition to regulating the output of simulated genes, supercoiling-dependent feedback tunes the size and frequency of transcriptional bursts. To investigate how these tunable parameters may affect synthetic gene circuits, we applied our model to a canonical gene circuit, a synthetic toggle switch constructed with different syntaxes. We find that circuit syntax affects the stability of states and sets biochemical parameters required for bistability, including repressor cooperativity and RNA stability. Finally, we explored how DNA supercoiling might support transcriptional coordination within the native genome of zebrafish (*Danio rerio*) to enable somite segmentation. We find that DNA supercoiling acts as a mechanism for coordinating expression between divergently expressed genes in the segmentation network. Supercoiling-dependent feedback supports tight regulation of these proximal segmentation genes, providing a molecular mechanism for the precise coordination of gene expression observed during somite formation (Zinani et al., 2021). Thus, supercoiling-mediated feedback represents a testable regulatory mechanism that can both explain native behaviors and guide synthetic designs.

## RESULTS

Simulating the behavior of native and synthetic circuits under the influence of transcription-induced feedback requires a model that integrates explicitly modeled RNA polymerase (RNAP) motion and RNA- and protein-mediated feedback mechanisms. Our method combines three modeling levels: an ordinary differential equation system that simulates the continuous progression of polymerases loaded onto DNA, a core stochastic system that models supercoiling-dependent polymerase initiation, and a user-specified stochastic layer that allows for simulation of other modes of transcriptional regulation such as the promoter-repressive and -activating interactions that are often included in synthetic circuits (see STAR Methods for details on model development).

**Figure 1.**
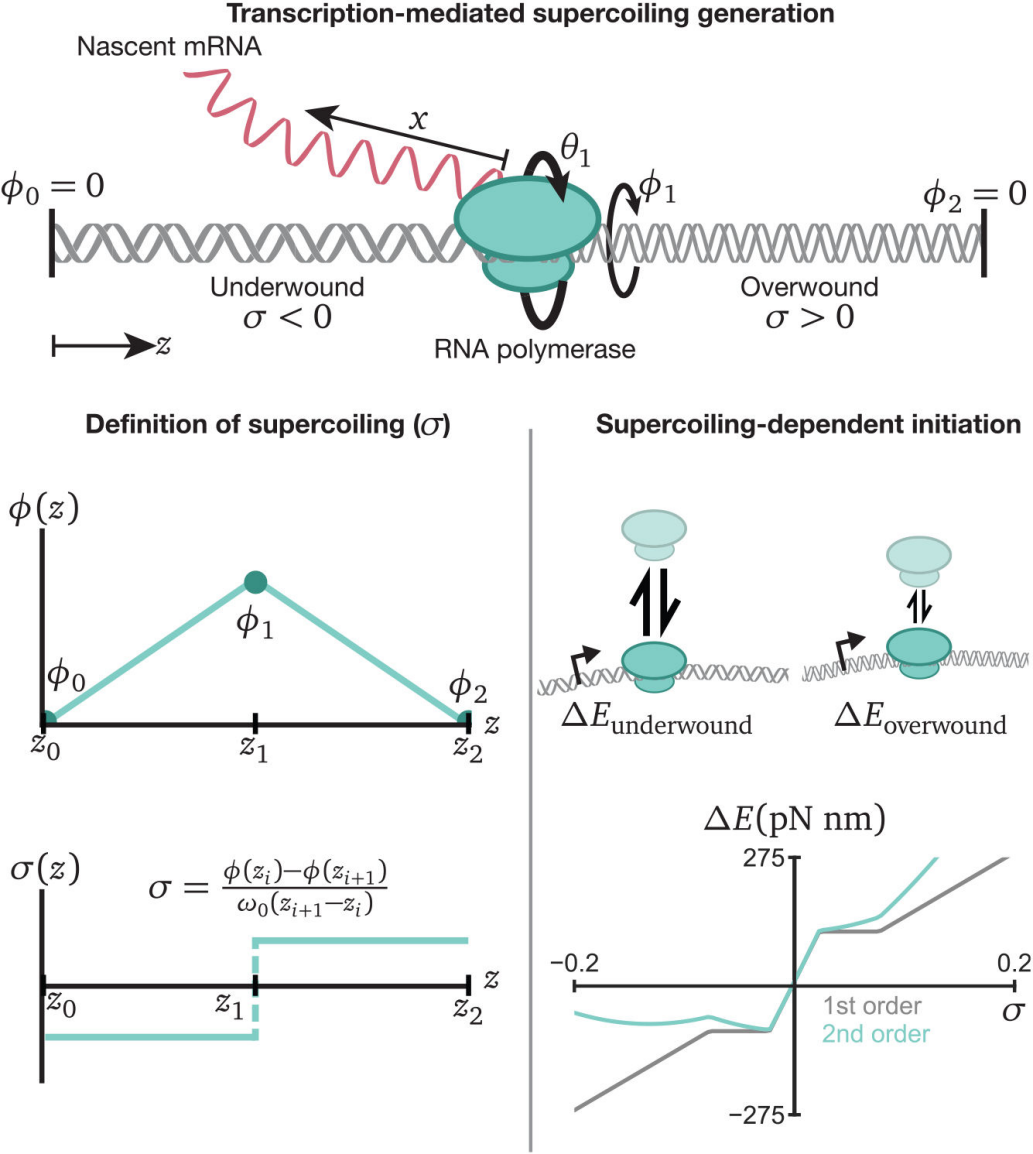
Four key variables define the location of each polymerase: its linear distance *z* along the genome, the length of the nascent mRNA transcript *x*, the rotation of the polymerase Θ, and the local DNA excess twist φ The relaxed DNA twist frequency *ω*_0_ has value *ω*_0_ = 1.85 radians/nm. The tradeoff between RNAP rotation and DNA rotation generates supercoiling upstream and downstream, with the drag generated by the nascent mRNA primarily balancing the torque caused by generated supercoils. In the limit of fast supercoiling relaxation relative to polymerase motion, the supercoiling density is constant in the region between polymerases and can be calculated from the slope of the linearly interpolated *φ*(*z*) graph. Using an energy model responsive to local supercoiling, we can derive supercoiling-dependent initiation terms to model differential polymerase loading rates.

As RNA polymerases move along chromatin, positive supercoiling is generated ahead of the polymerase and negative supercoiling is generated behind. Supercoiling generation occurs because the drag of the nascent mRNA rotating with the polymerase is balanced by torque arising from the over- and under-winding of the DNA (Figure 1). On the length scale of tens of kilobases, supercoiling diffusion is negligible, causing the supercoiling density, σ, to take on a constant value between polymerases. Here, we model supercoiling as affecting gene expression in two ways: excessive supercoiling can both stall polymerases due to excess torque and enhance or suppress polymerase binding at promoter regions due to supercoiling-dependent initiation (Figure 1; STAR Methods).

### Gene syntax and boundary conditions define DNA supercoiling dynamics, expression profiles, and noise

**Figure 2.**
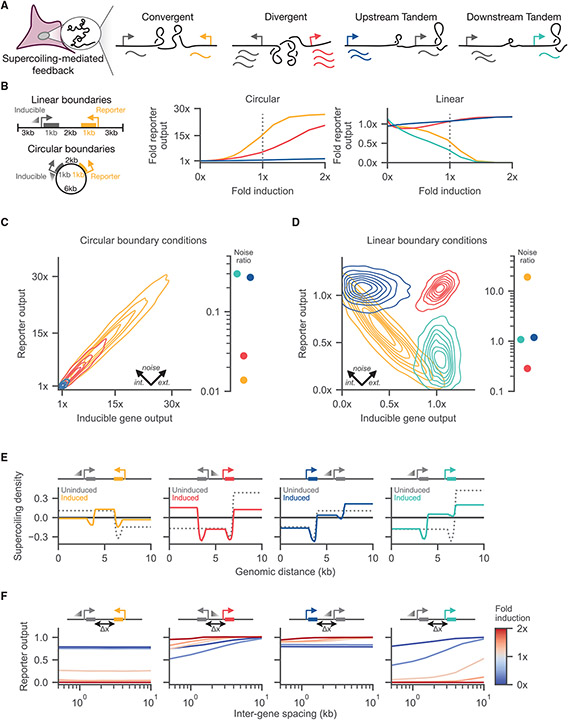
Supercoiling-dependent feedback induces syntax-specific expression profiles (A) Two-gene circuits serve as a testbed for investigating supercoiling-mediated feedback. All four syntaxes include a reporter gene (colored) and an inducible gene (gray). (B) Two classes of boundary conditions are simulated. Linear boundary conditions are simulated with adjacent “walls” that prevent supercoiling propagation, whereas circular boundary conditions allow supercoiling generated at one gene to freely affect other genes in either direction around the circle. At right, mean reporter expression is plotted as a function of the level of induction of the adjacent gene for circular and linear boundary conditions. Reporter output is normalized to the uninduced expression case by dividing mRNA counts by a constant value per boundary condition case (10 mRNAs for the circular case and 250 mRNAs for the linear case). (C) Gene expression distributions of simulations with circular boundary conditions are shown, where the adjacent induced gene is equally induced relative to the reporter gene. For each of the four syntaxes, the expression variance can be decomposed into intrinsic and extrinsic noise components; the ratio of intrinsic to extrinsic noise is shown on the right. Reporter output is normalized by dividing mRNA counts by a constant value (10 mRNAs). (D) Gene expression distributions and the intrinsic to extrinsic noise ratios are shown for linear boundary conditions. Reporter output is normalized by dividing counts by a constant value (250 mRNAs). (E) The mean supercoiling density of linear constructs are shown as a function of induction of the inducible gene. Induction (colored line) displays syntax-specific behavior compared with the uninduced case (dashed line). (F) Reporter output is shown as a function of inter-gene spacing (Δ*x*) at five different induction levels. Reporter output is normalized by dividing mRNA counts by a constant value (250 mRNAs). See also Figure S1.

In order to characterize the behavior of supercoiling-mediated feedback, we simulated a series of two-gene systems. These experimentally accessible circuits allow us to test and understand the core design considerations—syntax, relevant boundary conditions, and other experimentally tunable parameters—within a well-defined and controlled system. Our two-gene systems consist of a reporter gene that is constitutively active and an adjacent, inducible gene placed in either a tandem orientation with the reporter upstream, tandem orientation with the reporter downstream, convergent orientation, or divergent orientation (Figure 2A).

Varying syntax, we examined how boundary conditions affect the expression profiles of the reporter and inducible genes. The type of boundary condition determines how transcriptionally generated supercoiling propagates to adjacent genes. Experimentally, plasmid constructs and genomically integrated cassettes allow for interrogation of circular and linear boundary conditions, respectively. Plotting reporter expression as a function of adjacent gene induction, we observed that circular boundary conditions show a monotonic increase in reporter output that scales by syntax (Figure 2B). With circular boundary conditions, both the convergent and divergent syntaxes show a large enhancement of expression relative to the uninduced case, whereas the tandem cases show only a slight enhancement. This phenomenon occurs because, on a circle, the convergent and divergent syntaxes differ only in the relative lengths of the intergenic and inter-promoter regions; as such, negative supercoiling density will accumulate in the inter-promoter region, enhancing expression in the convergent and divergent syntaxes. In contrast, linear boundary conditions show diverse behaviors. The upstream-tandem and divergent syntaxes maintain high expression levels, while the convergent and downstream-tandem syntaxes show decreasing reporter expression with increasing induction of the neighboring gene.

Transcriptional noise substantially contributes to variance of gene expression (Quarton et al., 2020), often confounding circuit designs. Thus, designing circuits to respond to or suppress noise may improve circuit performance. To investigate how syntax affects expression profiles and noise, we simulated the ensemble behavior for circular and linear two-gene systems at equal transcriptional induction (gray dotted line in Figure 2B). To examine different forms of noise, we decompose the population variance into an extrinsic noise component that describes how “all” genes co-vary within a cell and an intrinsic noise component that describes the inter-gene variance within a cell. Then, we define the noise ratio as the intrinsic noise divided by the extrinsic noise. For the circular-boundary condition simulations, we found that all four ensembles are dominated by extrinsic noise (Figure 2C). In contrast, while the tandem syntaxes with linear boundary conditions show approximately equal intrinsic and extrinsic noise, the linear convergent and divergent populations showed diverging shifts in the variance distribution (Figure 2D). Despite similar levels of extrinsic noise, the divergent syntax minimizes intrinsic variation between the two genes while the convergent syntax maximizes inter-gene variation. Moving forward, we used the linear set of boundary conditions to analyze system behaviors as we observe the richest set of behaviors under these boundary conditions.

To understand the mechanisms that support syntax-specific expression profiles (Figure 2D), we examined the ensemble supercoiling density of our two-gene systems. Putatively, differences in supercoiling across the two-gene systems give rise to differences in RNAP initiation and thus affect gene expression. To observe supercoiling across the two-gene systems, we averaged the supercoiling density across the simulated ensemble. To examine how induction of the adjacent gene changes supercoiling density, we compared the profiles for when the adjacent gene is uninduced (0-fold induction) and induced (1-fold induction). As expected, the uninduced cases uniformly show that positive supercoiling accumulates downstream of the constitutively active reporter gene while negative supercoiling accumulates upstream (Figure 2E; for other induction levels, see Figure S1A). Differences in basal expression of the reporter correspond to differences in supercoiling profile across the different syntaxes. While each uninduced case generates an equal magnitude of positive and negative supercoiling upstream and downstream, the distance from the reporter gene to the boundaries differs depending on syntax, leading to the shorter segment having the higher magnitude of supercoiling density. Upon induction of the adjacent gene, supercoiling accumulates within the intergenic regions in a syntax-specific manner.

Supercoiling density determines both supercoiling-dependent initiation and polymerase stalling. Changing the inter-gene spacing directly tunes the transcriptional activity required to reach a specified supercoiling density and thus reach different expression profiles. To understand the spacing-driven deviations in reporter behavior, we simulated our linear two-gene circuits with different inter-gene spacings from 500 bp to 10 kb and plotted the reporter output of each circuit (Figure 2F). We found that the convergent and tandem-upstream syntaxes show minimal spacing-dependent changes in reporter output; the reporter output in these two syntaxes is mostly dependent on the level of adjacent induction. In contrast, the reporter output in the divergent and tandem-downstream syntaxes depends on inter-gene spacing. With weak adjacent induction in the divergent case (blue curves), decreasing the inter-gene spacing *decreases* reporter output. We attribute this decrease to the effect of polymerase stalling; a small inter-gene spacing increases the local supercoiling density which can lead to polymerase stalling. At high adjacent induction (red curves), strong supercoiling-driven polymerase initiation overcomes the stalling effect, leading to a relatively distance-insensitive divergent reporter output. Finally, in the tandem-downstream syntax, in addition to the strong suppression of reporter output at high adjacent induction, a short inter-gene spacing further reduces reporter output by increasing the supercoiling density. Thus, depending on syntax, supercoiling-dependent feedback can be tuned by changing inter-gene spacing.

### DNA supercoiling dynamics confer rapid, tunable coupling between adjacent genes

**Figure 3.**
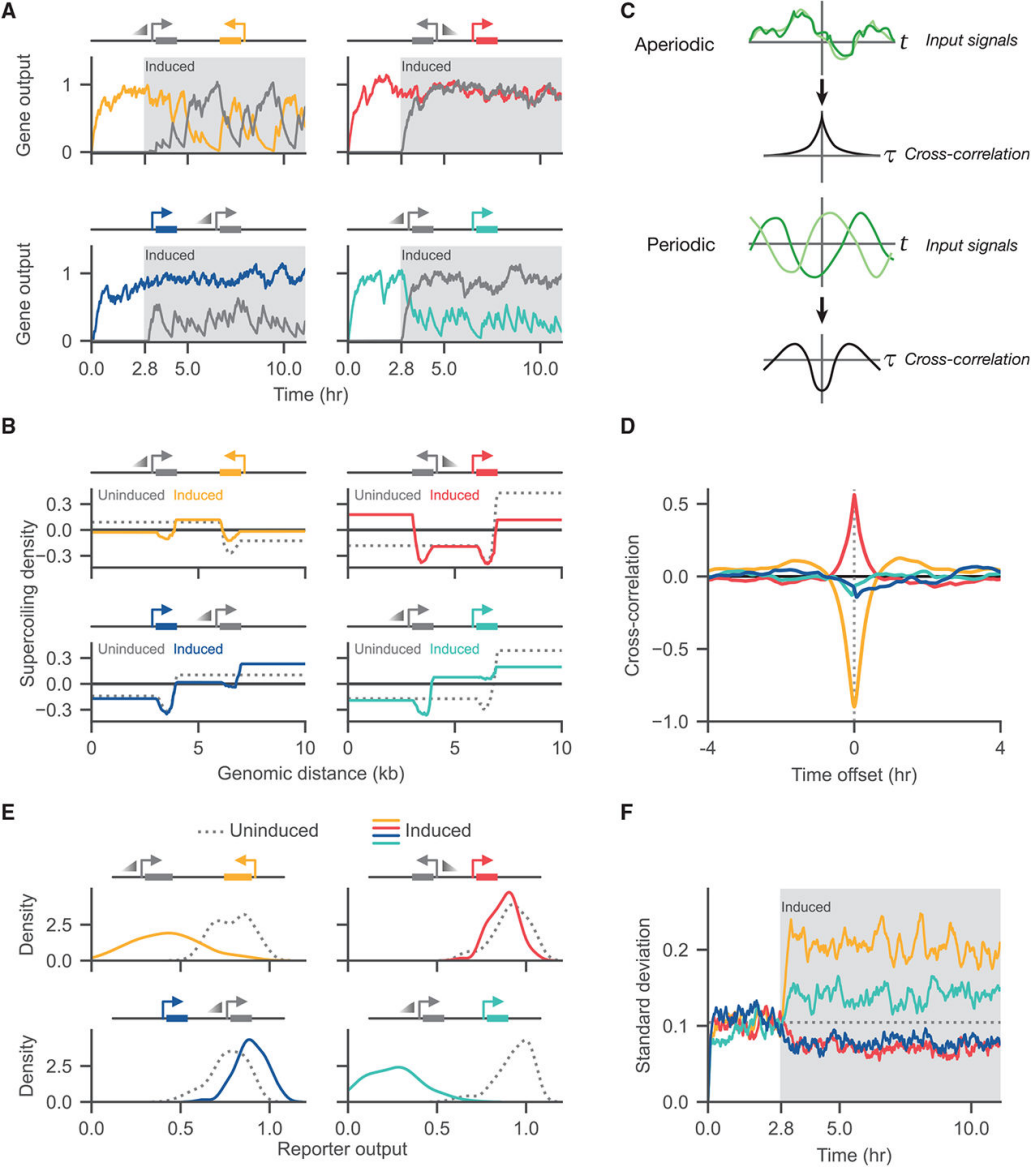
Supercoiling-dependent feedback induces dynamic coupling and altered variance between genes (A) mRNA counts over time from example individual simulations for the four syntaxes are shown. Simulations are initialized with only the reporter gene (colored) active, with the adjacent gene (gray) enabled with equal basal expression after 10,000 s (2.8 h). (B) The average ensemble supercoiling density is shown both before and after adjacent gene induction. (C) The cross-correlation of two signals *f*(*t*), *g*(*t*) at a time offset *τ* can be calculated by “sliding” one mean-centered signal relative to the other mean-centered signal and integrating the product of the resulting signals. (D) The cross-correlation between the two genes is shown for the equal-induction case across the four syntaxes. The convergent and divergent syntaxes showed the strongest cross-correlation, with the convergent case showing periodic behavior and the divergent case showing strong correlated expression. (E) Distributions of the reporter output before (dotted) and after (solid) induction of the adjacent gene show changes in both the mean and standard deviation due to adjacent expression. (F) Ensemble noise behavior for the four simulated syntaxes is shown by plotting the standard deviation of the reporter gene across the ensemble of simulations as a function of time. See also Figure S1 and Videos S1, S2, S3, and S4.

To explore the impact of DNA supercoiling beyond the ensemble steady-state behavior, we investigated the dynamic behavior of our system. To understand the initiation dynamics of the system, we initialized two-gene constructs with only the constitutive reporter gene active. After a settling period (2.8 h), we induced transcription of the adjacent gene (Figure 3A). We observe that expression dynamics vary extensively by syntax. In the convergent syntax, we observe anti-correlated dynamics, with “either- or” promoter activity (Video S1). In contrast, the divergent syntax supports high levels of expression from both genes (Video S2). When comparing the two tandem orientations, we observe strong biasing of expression toward the upstream gene with stochastic bursts of expression from the downstream gene (Videos S3 and S4). While this upstream dominance does not completely disable the downstream gene, activation of the upstream gene reduces the average expression of the downstream reporter.

To ensure that this observed behavior was not an artifact of enabling adjacent induction later in the simulation, we need to verify that the two-gene ensembles are ergodic (not kinetically trapped or otherwise dependent on initial condition). To start, we examined the ensemble expression distribution at the simulation endpoint (Figure S1B), and found that we recapitulate the population behavior observed in Figure 2D. As an additional check, we plotted the ensemble-averaged supercoiling density both before (at 2.3 h) and after (at 11 h) adjacent gene induction (Figure 3B). If the system is ergodic, then the ensemble behavior observed before and after the adjacent gene is turned on (snapshots of one population separated in time) should match ensemble behavior of simulations where the adjacent gene is time-independently active or inactive (snapshots of separate populations). Figure 3B does replicate the behavior observed in Figure 2E, indicating that the two-gene circuit populations are ergodic.

To expand our understanding of the emergent supercoiling-dependent dynamics, we examined the temporal correlation of transcription between both genes. To quantify the correlation and extract temporal patterns, we computed the normalized cross-correlation between the gene outputs following induction. The normalized cross-correlation of two signals is itself a function of a time offset; the normalized cross-correlation at some offset time *τ* can be thought of as the Pearson correlation coefficient between the two signals where one has been shifted by *τ* (Figure 3C; see "Computing cross-correlation" in STAR Methods for more detail). In particular, periodic but out-of-phase signals appear as strong negative and positive peaks on a cross-correlation plot. The time offset of peaks corresponds to the phase offset between the signals.

The four syntaxes show starkly different cross-correlation behaviors (Figure 3D). The convergent system shows a large anti-correlation at zero time offset with positive correlation peaks at offsets around ± 2 h. This combination suggests a periodic but out-of-phase behavior between the two genes with a period of around 2 h, confirming that our ensemble behaves similarly to the example simulation in Figure 3A. In contrast, the divergent syntax shows a strong positive peak at zero time offset, showing strong aperiodic, but correlated, behavior. The tandem syntax shows weak correlation between genes.

We then examined how adjacent induction affects the reporter output distributions. Visualizing the entire ensemble reporter output distribution per condition in Figure 3E at equal induction, we observe that adjacent induction changes both the mean and variance of the reporter output. In particular, the widths of the distributions change before and after induction, suggesting a change in the noise profile. At high adjacent induction, we observe that the upstream-tandem and divergent cases show enhanced transcription, with the downstream-tandem and convergent cases effectively turning off (Figure S1C).

As noise affects the properties of native and synthetic gene networks, we quantified the width of the these distributions by plotting the standard deviation of the ensemble reporter output as a function of time (Figure 3F). Prior to induction of the second gene, all four systems display similar standard deviations. Syntax differences emerge upon induction of the second gene. We found that syntax strongly modulates the noise behavior of the reporter. In particular, the downstream-tandem and convergent syntaxes show a strong *increase* in noise levels while the upstream-tandem and divergent syntaxes show a small *decrease* in noise levels. These changes in noise may results from differences in transcriptional burst sizes and frequencies.

### Burst dynamics vary in different models of DNA supercoiling

**Figure 4.**
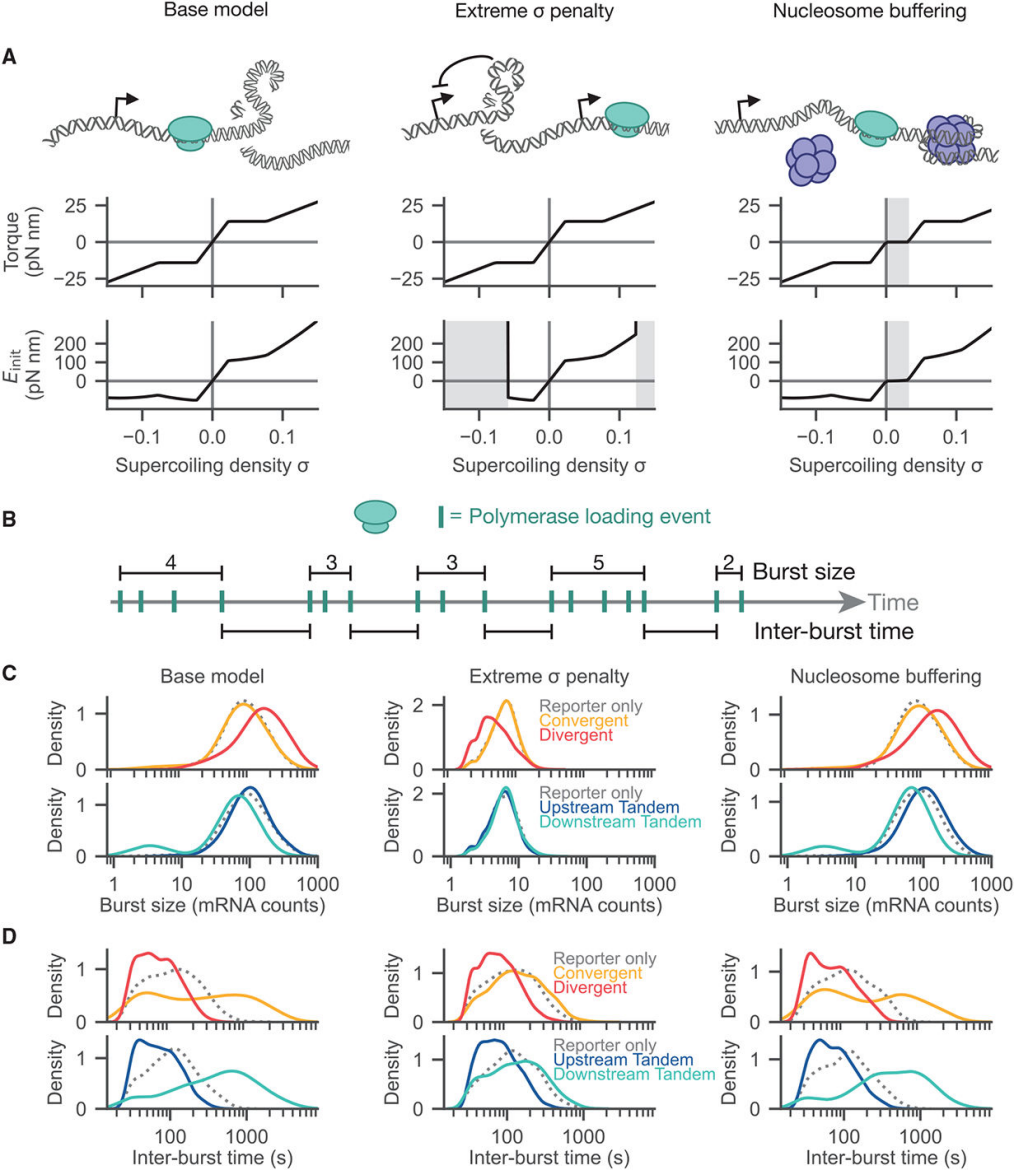
Burst dynamics vary in different models of DNA supercoiling (A) Two model perturbations are compared with the base model, one where polymerase initiation is impossible once the supercoiling density is extreme, and one where nucleosomes provide buffering against positive supercoiling. Each model tunes either the function relating torque and supercoiling density (middle) or the polymerase initiation energy function (bottom); changes relative to the base model are marked with a gray background. (B) Bursts are defined as a group of consecutive polymerase loading events. The size of a burst is defined as the number of loaded polymerases, whereas interburst time is defined as the gap between successive bursts. (C) The ensemble distribution of burst size is shown for the different orientations for each of the polymerase conditions. (D) The ensemble distribution of inter-burst time is shown for the different orientations for each of the polymerase conditions. See also Figures S1-S3.

Transcription occurs in bursts of activity, and native and synthetic mechanisms can modify burst dynamics (Desai et al., 2021; Chong et al., 2014; Popp et al., 2021). In our base model, transcriptional bursting arises from the stochastic addition of polymerases. We hypothesized that burst dynamics may explain the distribution dynamics observed in Figures 3E and 3F. While our base model provides a species-agnostic approach to investigate supercoiling-mediated feedback, the mechanical regulation of eukaryotic chromatin may introduce additional complexity and tunability to bursting dynamics. For example, nucleosomes can serve as a reservoir of negative supercoiling by stabilizing the wrapped double-loop of DNA (Le et al., 2019). Furthermore, at hypernegative or hyperpositive supercoiling densities, local chromatin structure may be disrupted causing structures such as R-loops and G-quadruplexes to preferentially form. These structures can block polymerase binding and motion (Stolz et al., 2019). Even when including our second-order correction term as detailed in Equation 7, our base model predicts favorable supercoiling-dependent polymerase initiation at the hypernegative value of *σ* = − 0.1 (Figure 4A). To address these complexities, we implemented two alternate models that extend our torque-response and polymerase-initiation energy equations.

To simulate the effects of structures forming at hypernegative or hyperpositive supercoiling densities, we penalize polymerase initiation by adding a near-infinite positive energy penalty to polymerase initiation at hypernegative and hyperpositive supercoiling densities (Figure 4A) roughly matching the density at which structures such as R-loops form (*σ* < – 0.06, *σ* > 0.125; Stolz et al., 2019). Putatively, these barriers enable us to simulate the formation of stable structures within the DNA that strongly penalize RNAP binding while remaining agnostic to any specific molecular structure. We find that penalization of extreme supercoiling globally reduces supercoiling density (Figure S2A) but maintains the qualitative syntax-dependent behavior of the steady-state, linear systems. However, syntax-specific differences are mostly eliminated for circular templates (Figures S2B and S2C).

To model nucleosomes, we updated our torque-response function to phenomenologically match *in vitro* experimental measurements (Le et al., 2019). Nucleosomes putatively “buffer” the effects of positive supercoiling by unbinding and releasing stored negative supercoiling. We accounted for this buffering by extending a zero-torque plateau within the region 0 ≤ *σ* ≤ 0.031 (Figure 4A). We found that the nucleosome buffering does not significantly affect the steady-state expression profile or supercoiling density observed in Figure 2 (Figures S3A-S3C).

With these three models, we simulated the four linear syntaxes and recorded polymerase initiation events per gene. We then examined the distribution of both burst size, which we define as the number of polymerase initiation events during a burst, and inter-burst time, the amount of time separating two consecutive bursts for the reporter gene (Figure 4B; see "Burst threshold choice" in STAR Methods for more detail). Upon induction of the adjacent gene, we find burst dynamics differ by syntax (Figure 4C). In both the base model and the nucleosome-buffering model, the downstream-tandem and convergent syntaxes show a reduced burst size compared with the divergent and upstream-tandem syntaxes. We attribute this reduction in burst size to accumulated positive supercoiling at the site of the reporter gene’s promoter. In contrast, induction of the adjacent gene in the divergent syntax increases the burst size of the reporter gene, putatively due to enhanced loading of polymerases facilitated by accumulated negative supercoiling. With penalization of hypernegative supercoiling density, the divergent syntax shows a decrease in burst size, potentially due a shift in the tradeoff between polymerase stalling and supercoiling-dependent initiation.

Examining the inter-burst time distributions, induction of the neighboring gene shifts the upstream-tandem and divergent syntaxes to shorter inter-burst times (Figure 4D). The down-stream-tandem and convergent syntaxes shift to longer inter-burst times upon induction of the adjacent gene. Interestingly, this qualitative observation holds independently of the choice of torque-energy model, suggesting that changes in inter-burst time—and its inverse, burst frequency—is a syntax-dependent effect that is largely insensitive to additional chromatin perturbations.

From these observations, we can now understand the population-level behaviors observed in Figures 3E and 3F. The increase of noise and reduction of mean expression value in the down-stream-tandem and convergent cases occurs concomitant with an increase in inter-burst time (Figure 4D). This is expected; if bursts occur rarely, stochastic fluctuations will have an outsized effect on each individual simulation, enhancing population variance. In contrast, the decrease of noise in the upstream-tandem and divergent syntaxes is matched by a decrease in the inter-burst time (Figure 4D). Because bursts happen more frequently, the ensemble reporter output is more stable as we approach the limit of constant transcription. Taken as a whole, syntax provides a powerful design parameter for inducing and tuning time-dependent behaviors between genes and shaping output gene distributions.

### Optimizing toggle-switch performance and stability through circuit syntax

From oscillators to pulse generators, synthetic circuits aim to precisely coordinate dynamic patterns of gene expression. However, emergent dynamics, mediated through DNA supercoiling, may support or impede the performance of dynamic circuits. To examine how supercoiling-mediated feedback influences a dynamic circuit, we applied our model to the classic repressor-mediated toggle switch (Gardner et al., 2000). Toggle switches are well characterized both theoretically (Gardner et al., 2000) and experimentally (Gardner et al., 2000; Yeung et al., 2017; Zhu et al., 2021). The behavior of a simple toggle under the additional influence of supercoiling-mediated feedback provides an ideal testbed to understand how syntax influences circuit performance.

**Figure 5.**
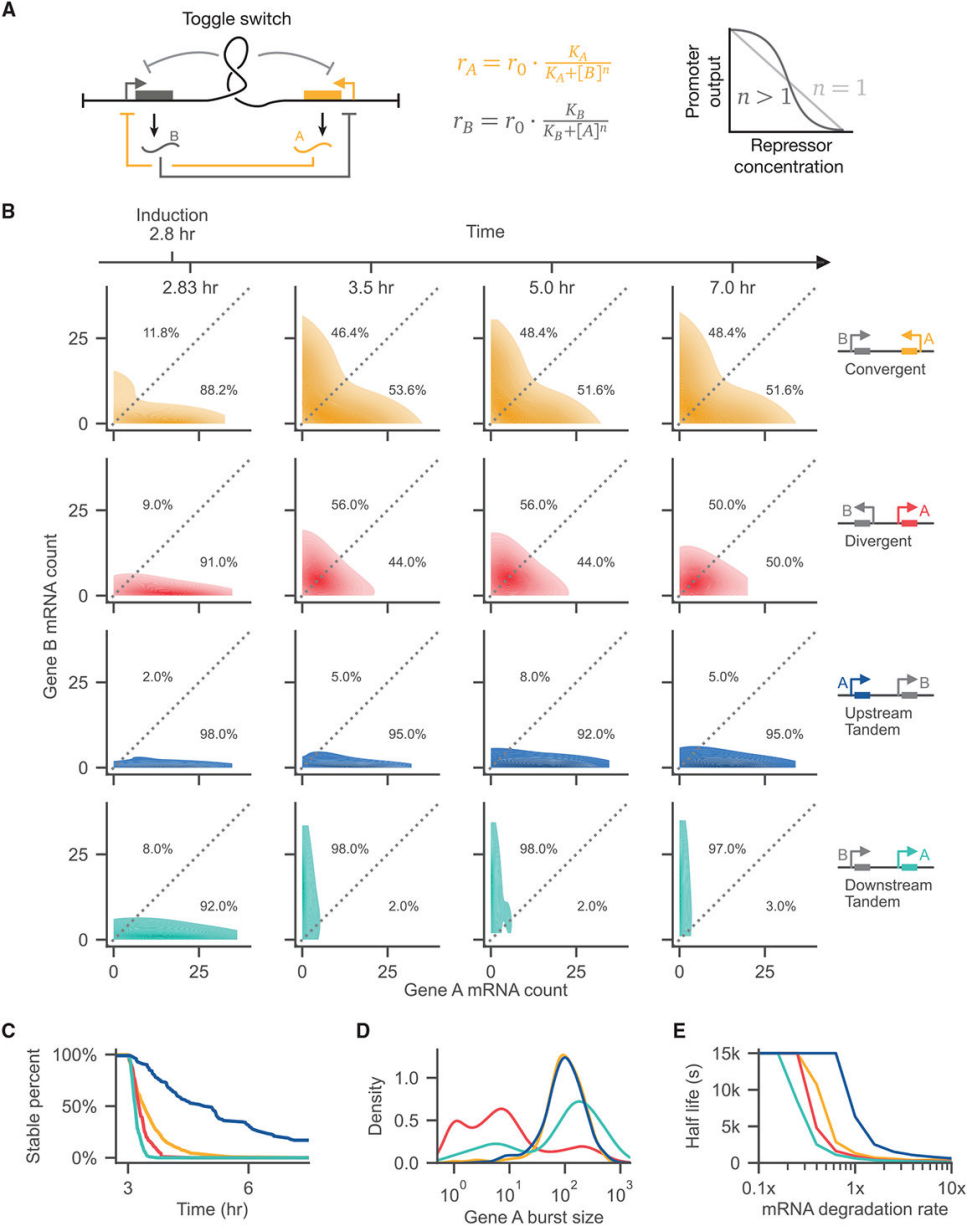
Toggle switches implemented as a mutually inhibitory pair of genes show syntax-specific stability (A) Schematic of a synthetic toggle switch composed of mutual transcriptional repressors, A and B, which are expressed from a promoter negatively regulated by the opposite gene. Repression follows a Hill function (center), which shows cooperativity based on the value of *n*. Reactions where *n* is greater than 1 show cooperativity. Simulated toggle switches are regulated both by a mutually inhibitory interaction at the mRNA level and via supercoiling-dependent phenomena. (B) The ensemble mRNA count distributions are shown as a function of syntax at four selected time points. All plots represent simulations where the Hill coefficient has been set to *n* = 2.0. (C) The stability, measured as the percentage of simulations in the ensemble that have never escaped the initial starting basin, of the four starting states of the system plotted as a function of time. (D) Expression burst size distributions of the initially active gene A are plotted as a function of circuit syntax. (E) The half-life at different values of the mRNA degradation rate are shown. As the mRNA degradation rate principally sets the average number of mRNA molecules, high degradation rates lead to systems with low overall mRNA concentration and concordant stochastic instability. See also Figure S4.

A toggle switch can be constructed with two genes that mutually repress each other (Figure 5A). Ideally, such a toggle switch exhibits bistability, generating two stable basins. If modeled with continuous, noise-free equations, a toggle switch will remain within one of the basins based on the initial conditions (Gardner et al., 2000). However, if we treat the mRNA concentration discretely with a stochastic simulation, the system escapes the stable basin with a certain probability, depending on the size of fluctuations relative to the steady-state values. How does supercoiling-mediated feedback affect this probability of escape? How might supercoiling-mediated feedback interact with mutual transcriptional repression and alter toggle-switch behavior? To answer these questions, we simulate the behavior of a two-gene toggle switch with our model for various circuit syntaxes. To establish the conventional dual repressor system used for toggle switches, we abstracted the regulatory interaction of the repressors using a Hill function to define the base promoter initiation rates:

(Equation 1) $$r_{A}=r_{0}\frac{K_{A}}{K_{A}+[B{]}^{n}}\;\;r_{B}=r_{0}\frac{K_{B}}{K_{B}+[A{]}^{n}}$$

for *r*_0_ = 1/160 s^−1^ and *n* = 2.0. Here, we do not explicitly model protein production. Rather, transcriptional repression directly depends on the mRNA counts of repressors. This parsimonious model allows us to understand the behavior of the system without introducing additional rate constants. We chose the half-max value *K*—the mRNA count at which the promoter activity is half that of *r*_0_ —to approximately match the mean steady-state value of either stable state to ensure that the toggle switch operates in the regime of maximum sensitivity (see "Toggle-switch parameter selection" in STAR Methods for details). In order to compare the behavior of the toggle switches, we initialized toggle switches of different syntaxes within one of the stable basins (gene A) and induced the second gene (gene B) after 2.8 h (see Figure S4A for example runs). The system then evolves under simultaneous mutual inhibition from expression of the repressors as well as from supercoiling-dependent feedback.

Circuit syntax specifies unique toggle-switch dynamics that can be understood by visualizing the distribution of mRNA counts over time (Figure 5B). Initially, nearly all of the simulations in the ensemble lie along the axis corresponding to the initially active gene, gene A. As time progresses, each ensemble approaches and fluctuates around an equilibrium. The convergent syntax approaches an equilibrium where approximately half of the population distributes into each state. In this syntax, activation of either gene causes positive supercoiling to accumulate in the intergenic region, enhancing negative feedback between genes and thus between states. With divergent syntax, the toggle distributions converge toward monostability with low differential expression of either gene. Accumulation of negative supercoiling between genes enhances polymerase loading, weakening negative feedback. Finally, the vast majority of the simulations in the tandem syntax remain in or transition to the upstream-active state, demonstrating upstream dominance. We find that these results qualitatively hold for varying values of *n*, the repressor cooperativity coefficient (Figure S4E). We find that even in the absence of cooperativity, *n* = 1.0, the convergent syntax shows some level of bistability (Figure S4D), indicating that supercoiling-mediated feedback introduces a degree of nonlinearity that can reinforce toggle-switch function.

To quantify these distribution results, we computed the stable fraction of the ensemble, defined as the fraction of simulations that have never left the initial starting basin at a certain simulation time. The stable fraction monotonically decreases toward zero with time, as simulations that cross into the other stable basin are no longer counted as stable even if they return to the original basin. We observe substantial syntax differences in the dynamics of the stable fraction of the ensemble (Figure 5C). While the tandem orientations represent the extremes of stability, the convergent and divergent syntaxes exhibit intermediate stabilities. As expected, different burst dynamics characterize toggle-switch behaviors, varying by syntax. In particular, we observe that the divergent syntax displays *reduced* burst size compared with the other syntaxes (Figure 5D). In contrast, the inter-burst time distributions do not significantly vary with syntax (Figure S4C), suggesting that burst size, not burst frequency, is a key parameter tuned by supercoiling-mediated feedback in this context. We hypothesize that the divergent toggle switch may be governed by a conflicting interaction at the promoter level between supercoiling-mediated feedback and mutual inhibition. Overall, these trends suggest that the observed toggle-switch behavior emerges through correlated (or anti-correlated) transcription and changes in burst size, suggesting that toggle-switch behavior could be tuned orthogonally by supercoiling-mediated feedback.

For any stochastic system, the steady-state number of molecules influences the stability of the system. As the reservoir of molecules grows larger, the size of fluctuations relative to the total concentration decreases. For toggle switches, we expect that, as the number of steady-state mRNA molecules grows, we should approach the theoretical, continuous solution that predicts that no state switching occurs. To examine this expectation, we modified the simulated mRNA degradation rate, scaling *K* as described in "Toggle-switch parameter selection" in STAR Methods, and plotted the resulting half-lives (Figure 5E). As the mRNA degradation rate goes to zero, we increase the reservoir size and observe that the half-life for all syntaxes approaches the simulation upper-limit on the half-life (Figure 5E). Interestingly, increasing mRNA degradation rates reduces the asymmetry in the half-lives between the tandem-upstream and tandem-downstream syntaxes. These results suggest that state switching increases as mRNA degradation rate increases as expected.

### DNA supercoiling tightly coordinates expression of proximal segmentation genes

**Figure 6.**
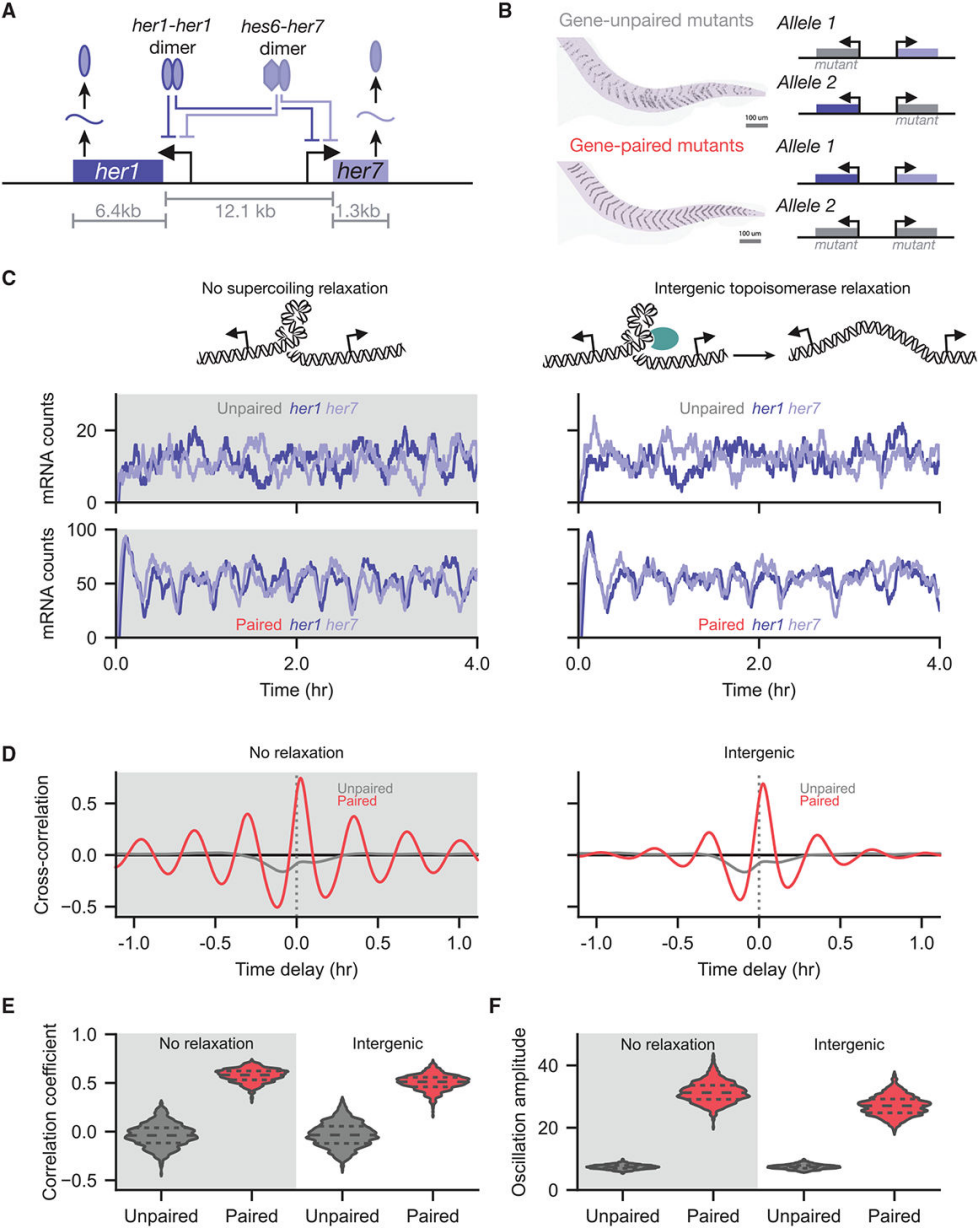
Supercoiling-mediated feedback supports robust transcriptional coordination within the zebrafish segmentation clock circuit (A) Schematic of the mutually inhibitory *her1-her7* system. Either a *her1-her1* dimer or a *hes6-her7* dimer can bind to either promoter, preventing transcription of the downstream gene. (B) Coupling between *her1-her7* genes on the same allele supports proper zebrafish somite formation (Zinani et al., 2021). Disruption of this intra-allele coupling through unpaired mutations in *her1-her7* leads to loss of proper segmentation. (C) Example simulations of *her1* and *her7* mRNA levels are shown as a function of gene syntax and topoisomerase activity. (D) The ensemble cross-correlation between the *her1* and *her7* mRNA counts is shown across pairing and topoisomerase conditions. The large maxima at *τ* = 0 combined with large roughly symmetric minima observed in the gene-paired cases signal the strong cyclic behavior observed experimentally in zebrafish. (E) The distribution of correlation coefficients between the *her1* and *her7* mRNA counts is shown for the various pairing and topoisomerase conditions. (F) The oscillation amplitude over the ensemble is shown for the various conditions. See also Figure S5.

DNA supercoiling provides a mechanism for the precise coordination of co-localized genes. Through colocalization, native circuits may incorporate transcription-linked feedback mechanisms to reduce noise and tune cell-state specific output in tightly regulated, dynamic processes such as somite formation. In zebrafish, proper somite segmentation requires precise coordination of two clock genes, *her1* and *her7. her1* and *her7* form an inhibitory feedback loop encoded in a divergent syntax (Figure 6A). Proper somite formation requires one intact allele of *her1* and *her7,* provided these genes are expressed from the same locus (Zinani et al., 2021). Mutant zebrafish embryos where *her1* and *her7* are only expressed from separate loci eliminate any supercoiling-mediated coupling while retaining the dimer-mediated inhibitory feedback loop. Zinani et al. (2021) found that, in the gene-unpaired embryos, transcriptional coordination between genes is lost and proper somite segmentation is disrupted (Figure 6B). Consequently, physical colocalization represents an important feature supporting transcriptional coordination between genes and proper somitogenesis, which may be mediated by supercoiling-mediated feedback.

Based on our above results from two-gene systems, we hypothesized the feedback from DNA supercoiling supports coordination between discrete transcripts expressed from divergent promoters. Using our full computational model, we replicated the previously developed stochastic reaction network. Importantly, *her1* and *her7* are regulated in a binary fashion; the promoters are either completely off when bound by a dimer or expressed at their basal rate when unbound. We simulated two cases: an *unpaired* system where the simulated genes were separated by a large distance (1 Mb) to prevent supercoiling interactions, and a *gene-paired* system where *her1* and *her7* were spaced at their genomically active locations. In the paired system, linear boundary conditions were used with boundaries chosen at the nearest adjacent genes on each side in the zebrafish genome.

Strikingly, the gene-paired case shows strong periodic levels of mRNA expression (Figure 6C). In fact, such levels of periodicity are not observed even in the original computational model presented by Zinani et al. (2021) (Figures S5A and S5B); we confirmed that this is not simply an artifact of the uniform time resampling performed in order to compare our model behavior to the literature model (Figure S5C). The level of periodicity appears sensitive to our second-order polymerase initiation model. Performing simulations with a weakened second-order penalty term (see STAR Methods; Figures 2 and S7) reduces the amount of periodicity observed (Figures S5D to S5G).

Examining the time-dependent nature of the *her1-her7* system, we plotted the ensemble cross-correlation for paired and unpaired genes (Figure 6D). Here, we found that, in addition to the enhanced positive correlation peak at a time delay of *τ* = 0 seconds, the paired case showed exceptionally strong, nearly symmetric cross-correlation at positive and negative time offsets. Such cross-correlation is the hallmark of a periodic signal. Thus, both individual examples (Figure 6C) and ensemble behavior (Figure 6D) show that supercoiling-mediated feedback provides a strong mechanistic driver of inter-gene coordination in the *her1-her7* clock circuit that is inaccessible to solely dimer-mediated regulation.

In order to confirm that these results apply across the ensemble, we examined the ensemble correlation between the counts of *her1* and *her7* mRNA (Figure 6E). We found that while the unpaired case shows minimal correlation, the gene-paired case shows strong correlation between the two clock genes. We attribute this strong, periodic correlation to the additional biophysical coupling conferred by the divergent syntax. Notably, we observed that our model predicts an increase in the amplitude of oscillations (Figure 6F). *In vivo,* loss of gene pairing reduces oscillation amplitude, leading to improper segmentation (Zinani et al., 2021).

Because this periodic behavior depends on biophysical coupling, we investigated whether the periodicity was robust to topoisomerase activity, nucleosome buffering, or hypernegative chromatin structure formation. We found that intergenic topoisomerase relaxation (see STAR Methods) does not abrogate the periodic behavior, with strong correlated oscillations still visible (Figures 6D-6F). However, intergenic topoisomerase activity does reduce the cross-correlation after a few periods, indicating that topoisomerase activity contributes to variance in oscillation frequency while still supporting strongly correlated *her1* and *her7* expression. Intragenic topoisomerase activity showed similar behaviors (Figure S5I). Finally, we found that while penalization of hypernegative supercoiling densities eliminates periodic behavior (Figures S2D to S2G), periodicity is maintained in the presence of nucleosome buffering (Figures S3D to S3G). Thus, we propose that supercoiling-mediated feedback offers a mechanism to support robust oscillations in the *her1* and *her7* network for proper somite formation.

## DISCUSSION

Transcription induces significant variance in gene expression. At a single-cell level, individual genes are expressed stochastically, with most genes experiencing relatively long periods of quiescence punctuated by bursts of polymerase activity. Phenomenological models of this process based on stochastic probability distributions can provide some insights, but defining the mechanically regulated physical factors that influence RNA polymerase dynamics will improve existing models of gene regulation and support enhanced design of transgenic systems. Importantly, we sought to develop a model that would define a set of experimentally testable predictions as well as lay the groundwork for future modeling across multigenic loci and circuits.

In this work, we developed a model of supercoiling-mediated feedback that captures emergent coupling between neighboring genes to influence expression levels as well as dynamics. This model allowed us to tractably compute polymerase activity at the scale of synthetic circuits (Figure 2). Within supercoiling-mediated feedback, we included both supercoiling-dependent polymerase motion terms and supercoiling-dependent polymerase initiation terms. This computational framework lays the groundwork for understanding how DNA supercoiling functions as a regulatory mechanism that can be integrated with canonical biochemical models of gene regulation. Using this model, we extracted insights into how mechanical and biochemical regulation combine to generate diverse profiles of expression and support or impede the performance of gene networks.

We find that induction of neighboring genes significantly influences the transcriptional activity of both genes (Figure 2). Syntax-specific differences in DNA supercoiling dynamics, expression profiles, and noise emerge due to physical coupling. We find that such a system regulated by this biophysical coupling is responsive to expression level changes in adjacent genes, with both mean expression and population variance changing as a function of gene orientation (Figure 3). The observed supercoiling-mediated feedback is itself dependent on inter-gene spacing, mRNA degradation rate, and other variables tunable in an experimental setting. Generally, accumulated negative supercoiling leads to correlated bursting, which occurs concomitant to a decrease in intrinsic noise. In contrast, accumulated positive supercoiling can lead to anti-correlated bursting, which instead enhances intrinsic noise. While the tandem syntax does not lead to large supercoiling accumulation in the intergenic region, we observe upstream dominance, where the upstream gene is more highly expressed than the downstream gene.

Our prediction of burst dynamics (Figure 4) complements theoretical and experimental investigations of cooperative interactions of RNA polymerases arising from the beneficial cancellation of positive and negative supercoiling generated by adjacent polymerases (Sevier and Hormoz, 2022; Kim et al., 2019). We predict that syntax causes a 2- to 3-fold change in burst size (Figure 4C) but can shift inter-burst time by an order of magnitude (Figure 4D). In addition, the syntax-specific trends in inter-burst time are not strongly affected by other potential mechanical regulators of eukaryotic chromatin—nucleosomes and strained structures such as R-loops—suggesting that syntax may robustly control gene expression through differences in burst dynamics. While we do not directly examine transcription elongation rates, we similarly predict syntax-specific differences in expression dynamics but observe distinct syntax-specific behaviors in our model (Sevier and Hormoz, 2022; Tripathi et al., 2021). We also find that intergenic distance only weakly affects supercoiling feedback (Tripathi et al., 2021). In alignment with experimental work, we find that positive supercoiling accumulates in the intergenic region of convergently oriented native genes (Guo et al., 2021). When combined with sequencing methods that precisely measure nascent mRNA transcription (Mellor et al., 2016), these methods may provide a window into experimental systems in order to test theoretical predictions of our work and others.

The fast timescale of supercoiling-mediated feedback offers access to a uniquely tunable and orthogonal form of gene regulation. In contrast to regulatory mechanisms dependent on relatively long timescales, such as mRNA- and protein-mediated systems, supercoiling-mediated feedback occurs at the timescale of seconds. Polymerases can stall and unstall each other within seconds, while local polymerase loading rates can vary over the course of minutes. By combining the fast dynamic feedback with slower classic feedback mechanisms, circuit regulation can be selectively stabilized or destabilized. We found that specification of syntax within a simple two-gene toggle switch generated diverse behaviors, including a reasonably stable switch, a hypersensitive toggle with hysteresis, and an asymmetric system that preferentially decays toward a single target state (Figure 5). As a rapid mechanism for coordinating transcriptional dynamics, supercoiling-dependent feedback may support intergenic coordination in native systems. Examining the zebrafish segmentation clock, we find that addition of supercoiling-mediated feedback recapitulates the synchronized, periodic expression of the clock genes, *her1-her7* (Figure 6).

Our model integrates supercoiling-mediated biophysical feedback with classic gene regulation motifs that are well studied in native and synthetic contexts. This unified framework brings us closer to an understanding of how supercoiling contributes to transcriptional regulation. We offer testable predictions about the performance of genetic circuits. The predicted changes in reporter output, supercoiling density, and burst dynamics observed in Figures 2-4 are experimentally accessible with modern sequencing and single-cell imaging technology (Guo et al., 2021; Mellor et al., 2016; Patel et al., 2022). Experimental verification of our theoretical results will aid in constructing a mechanistic understanding of how transcription-induced supercoiling couples expression. Harnessing these insights will enable gene regulation at the level of transcription, providing a robust method to control expression dynamics, levels, and noise.

### Limitations of the study

In deriving our model, we made several simplifying assumptions. Our derivation of the energy function for supercoiling-dependent polymerase initiation adds relevant molecular detail to our model. The second-order correction term reflects the asymptotic relationship observed in *in vitro* assays between torque and supercoiling density for underwound DNA (Le et al., 2019). Inclusion of this correction supports the periodic behavior of the native *her1-her7* clock circuit, suggesting this term may accurately capture regulation *in vivo.* For simplicity, we model the dynamic processes of RNAP binding, initiation, and pause release as a single reaction. In real biological systems, each of these processes may vary across the genome by sequence and by the presence of nucleosomes, transcription factors, and other DNA-binding proteins. Formation of supercoiled structures shows sequence bias *in vitro* that may affect *in vivo* structures and gene regulation (Kim et al., 2018).

While we do approximate the behavior of the system in the presence of hypernegative/hyperpositive chromatin structures and nucleosomes, we do not explicitly model the presence of these and instead perturb our torque and polymerase binding energy functions. This implicit simulation method may not accurately capture the discrete nature of these phenomena, especially in the case of positive supercoiling waves displacing nucleosomes. Furthermore, while real topoisomerases dynamically relax chromatin and are often recruited to sites of active transcription (Baranello et al., 2016), we use a simplified model that instantly relaxes DNA in a non-specific manner. We find that our systems are mostly insensitive to this simple model of topoisomerase activity; future work using a different topoisomerase activity model may reveal additional roles for supercoiling relaxation.

Our model also excludes polymerase collision, premature termination, and the impact of 3D structures and loop domains formed by protein complexes such as CCCTC-binding factor (CTCF) and other structural maintenance of chromosomes (SMC) proteins. More broadly, we assume that regions of simulated chromatin are uniformly accessible and have equal torque responses during polymerase elongation; these assumptions may fail at the boundaries of chromatin domains. Finally, we also neglect the speed of supercoiling diffusion. While this is expected to be a negligible effect at the scale considered in this work, supercoiling diffusion remains slow at the scale of hundreds of kilobases to megabases.

## STAR★METHODS

### RESOURCE AVAILABILITY

#### Lead contact

Further information and requests for resources, datasets, and code should be directed to and will be fulfilled by the lead contact, Kate E. Galloway (katiegal@mit.edu).

#### Materials availability

This study did not generate new unique reagents.

#### Data and code availability

- All unprocessed and preprocessed simulation data reported in this paper has been deposited at Zenodo and is publicly available with the DOI: https://doi.org/10.5281/zenodo.7041641.
- All original code has been deposited at Zenodo and is publicly available with the DOI: https://doi.org/10.5281/zenodo.7054394.
- Any additional information required to reanalyze the data reported in this paper is available from the lead contact upon request.

### METHOD DETAILS

#### Supercoiling-dependent transcription model

Supercoiling is defined as the amount of excess twist φ relative to relaxed DNA. Relaxed DNA rotates one full revolution per ≈ 10 basepairs (1bp ≈ 0.34 nm); thus its relaxed twist is *ω*_0_ = 1.85 radians/nm. Supercoiling density can generally be defined as a varying function of genomic location *σ*(*z*). However, in a region of constant supercoiling density, we can use the excess DNA twist *φ_i_, φ*_*i*+1_ at the endpoints *z_i_, z*_*i*+1_ to define the supercoiling density as:

(Equation 2) $$\sigma =\frac{\varphi_{i}-\varphi_{i+1}}{\omega_{0}(z_{i+1}-z_{i})}$$

For a polymerase with *φ*_1_ > 0 between endpoints with *φ*_0_ = *φ*_2_ = 0, Equation 2 implies that the supercoiling density is *positive* in front of the polymerase and *negative* behind the polymerase (Figure 1). Following on the work of Sevier et al. (Sevier and Levine, 2018), we assume that on the length scales of synthetic and native circuits of interest (*O*(≈ 10kb)), the supercoiling density is constant in all regions between polymerases and other barriers. Because supercoiling diffusion and plectoneme hopping (Loenhout and Dekker, 2012) occur at rates faster than transcription (supercoiling diffusion: $D\approx O\left(0.5\;\frac{{\text{kb}}^{2}}{s}\right)$ versus transcription rate: $v_{0}\approx 0.05\;\frac{\text{kb}}{s}$) (Muniz et al., 2021), the supercoiling generated by a polymerase will diffuse outward far more rapidly than polymerases can move. As in previous reported work, we make a pseudo-steady assumption for inter-RNAP supercoiling—assuming that Equation 2 holds between polymerases—over the relatively small (~ 10 kb) genomic distances considered in this work in order to simplify the resulting model.

How does transcription both drive the process of supercoiling generation and react to changes in local supercoiling? Under the assumption of supercoiling relaxation, each polymerase is defined by four variables—the one-dimensional genomic location of the polymerase *z_i_*, the length of the nascent RNA *x_i_*, the excess twist at the location of the polymerase *φ_i_*, and the rotation angle of the polymerase (Figure 1). Then, two governing equations define the motion of all polymerases (Sevier and Levine, 2018). First, we equate linear polymerase motion with the rotational motion required to track the DNA groove:

(Equation 3) $$\underset{\text{RNAP velocity}}{\underset{︸}{\omega_{0}\frac{dz_{i}}{dt}}}=\overset{\text{RNAP rotation}}{\overset{︷}{\frac{d\theta_{i}}{dt}}}+\underset{\text{supercoiling generation}}{\underset{︸}{\frac{d\varphi_{i}}{dt}}}$$

where the change in *θ_i_* represents polymerase rotation and the change in *φ_i_* represents local rotation of the DNA. The second equation provides a torque balance between DNA-mediated torques on the left hand side and torque caused by drag acting on the nascent RNA:

(Equation 4) $$\underset{\text{torque acting on RNAP}}{\underset{︸}{\tau (\sigma (z_{i},\varphi_{i-1},\varphi_{i+1}))}}+\overset{\text{supercoiling restoring force}}{\overset{︷}{\chi \frac{d\varphi_{i}}{dt}}}=\underset{\text{nascent RNA drag}}{\underset{︸}{\eta x_{i}^{n}\frac{d\theta_{i}}{dt}}}$$

To develop a final system of ordinary differential equations, we still must define the torque response function *τ*(*σ*) and the polymerase velocity function $\frac{dz}{dt}\;=\;v(\tau )$. With these two functions, Equations 3 and 4 can be solved as in Sevier and Levine (2018). Here, we use Marko’s torque-response model of supercoiling which accounts for the thermodynamic behavior of both non-buckled, twisted DNA and buckled, plectonemic DNA (see Equations (S1) and (S4) in supplemental section B) (Marko, 2007). The resulting *τ*(*σ*) function exhibits a phase transition, where the torque response is nearly constant at intermediate values of σ where the DNA is transitioning from a locally-twisted phase to a plectonemic-phase (Figure S6A).

For the velocity response of a polymerase experiencing a torque *τ*_*f*_ in front and *τ*_*b*_ behind, we model polymerase stalling as:

(Equation 5) $$v(\tau_{f},\tau_{b})=\frac{v_{0}}{\left(1+e^{(|\tau_{f}|-\tau_{s})∕\tau_{w}})(1+e^{(|\tau_{b}|-\tau_{s})∕\tau_{w}}\right)}$$

where the stall torque *τ_s_* = 12 pN nm and stall-width *τ_w_* = 3 pN nm define a sigmoidal stall-response curve. As shown in Figures S6E and S6F, our results are only weakly dependent on the specific choice of *τ_s_* and *τ_w_*. Importantly, our selected phenomenological term will stall polymerase motion if *either* the torque upstream or downstream exceeds the stall torque *τ_s_*. Some models choose a stalling equation that only stalls if the difference between the upstream and downstream torque exceeds a stall torque (Tripathi et al., 2021); we chose this form, reasoning that the DNA unwinding and rewinding process opposed, respectively, by upstream and downstream torque could independently stall. When simulated, the difference between these stalling models is small in practice; polymerases at the start or end of the burst encounter both high upstream and downstream torques *and* a high torque difference, whereas polymerases in the middle of a burst experience both lower adjacent torques and a lower torque difference.

Taking the above equations together, we can simulate the coupled motion of an arbitrary number of polymerases as a single coupled ODE system. We further examine different experimental systems by implementing different boundary conditions that allow us to simulate both plasmid systems and genomically-integrated systems (see Boundary conditions).

#### Supercoiling-dependent initiation model

While the described differential equation system can simulate polymerase motion, we need a way to model the addition of polymerases to simulated genes. A simple strategy is to assume a supercoiling-independent initiation rate and use a stochastic simulation method to randomly add polymerases to transcriptional start sites at a certain fixed rate. However, this simple model assumes that polymerases can bind equally well to initiation sites independent of local supercoiling, missing supercoiling-dependent binding dynamics (Revyakin et al., 2004). In order to include supercoiling in a polymerase initiation model, we relate the basal expression rate to a corresponding base energy term. We can then additively introduce extra energy costs for polymerase binding under different local supercoiling conditions. Under the approximation that the direct energetic cost of locally melting the DNA to fit in the RNAP groove dwarfs the relative change in unwinding energy caused by supercoiling, the majority of the energetic cost comes from inserting supercoiling ahead and behind the inserted polymerase. Under this assumption, the first-order supercoiling energetic correction can be written as:

(Equation 6) $$E_{\text{sc}}=1.2\cdot 2\pi \cdot \tau (\sigma )$$

Is this a good approximation? We can estimate the energetic cost of local melting, and find that neglecting local melting leads to a minor change in the resulting energy as seen in Figure S6B. A full derivation of Equation 6 is given in Methods S1.

While this first-order energetic term introduces much-needed behavior to the modeled system—where locally high positive supercoiling decreases the RNAP initiation rate and locally negative supercoiling increases the RNAP initiation rate—at extreme values of σ, this energetic term gives aphysical predictions. In particular, under highly negative supercoiling densities, the energetics of polymerase loading become increasingly favorable, with loading sometimes occurring more than two orders of magnitude faster when compared with relaxed DNA. To correct for this behavior, we add a second-order (quadratic) term that constrains polymerase loading at highly positive or negative local supercoiling:

(Equation 7) $$E_{\text{sc}}\;=\;1.2\cdot 2\pi \cdot \tau (\sigma )+\alpha \cdot \tau_{0}\cdot \sigma^{2}$$

for *τ*_0_, the relevant scale factor in the *τ*(*σ*) equation (Equation (S1) and (S3) from (Marko, 2007)) and α, a positive tunable parameter. As the *τ*(*σ*) equation is linear in σ outside of the phase-transition region, this added *σ*^2^ term can be contextualized as an additional term in the Taylor expansion of the physically-realistic *E*_sc_(*σ*) equation. This form of the binding energy enables us to qualitatively match the experimentally observed asymptotic behavior between torque and supercoiling for underwound DNA (Le et al., 2019).

For these three models of supercoiling-dependent initiation, we found that the supercoiling-independent initiation model predicted only small changes in reporter output (Figure S7A). Comparing the first- and second-order models, we found that a critical value of α existed, *α*≈0.2, above which the second-order model demonstrated emergent non-monotonic behavior (Figure S7). At low values of α, the second-order model behaves similarly to the first-order model, so we used *α* ≈ 0.025 for this work. Increasing α beyond this chosen value appears to scale down reporter output without qualitatively modifying behavior (Figure S7).

When simulating the ODE model, the rate of stochastic polymerase initiation, *r*_initiation_, varies continuously based on the local supercoiling density σ at the transcription start site as:

(Equation 8) $$r_{\text{initiation}}=r_{\text{base rate}}\cdot e^{-E_{\text{sc}}∕(k_{B}T)}$$

#### Additional discrete reaction model

Many of the native and synthetic systems of interest include mechanisms of gene regulation that rely on other regulatory species. In order to analyze these types of systems using our supercoiling model, we extended our model to simultaneously simulate arbitrary discrete stochastic equations—such as those commonly used in the literature to model protein production, degradation, dimerization, and more. This addition allowed us to model discrete events otherwise not accounted for in the continuous model.

Importantly, this framework allows us to simulate the activity of topoisomerases. While the total amount of supercoiling (the integral of supercoiling density) is conserved, topoisomerases can be modeled as stochastic events that redistribute supercoiling in certain regions. Here, we simulated the removal of supercoiling in either intergenic or intragenic regions. Removal of supercoiling in intergenic regions is performed by updating the rotation of polymerases on adjacent genes to make the intergenic supercoiling density zero. After relaxation of supercoiling in intragenic regions, polymerases on the gene experience a constant supercoiling density which conserves overall supercoiling. With the exception of the segmentation gene network simulations in Figure 6 and related supplemental figures, we found that inclusion of topoisomerase relaxation did not appreciably change the observed results. Thus, unless otherwise stated, simulations were performed without topoisomerase relaxation.

In addition, we allowed the base initiation rate of genes to vary as an arbitrary function of all species concentrations in the model (*S_i_*), such that Equation 8 becomes:

(Equation 9) $$r_{\text{initiation}}=r(S_{i})\cdot e^{-E_{\text{sc}}∕(k_{B}T)}$$

By combining discrete reactions with the ability to dynamically change polymerase initiation rates, we are able to simulate a wide range of phenomena. For example, a cooperative repressive interaction between some repressor protein *R* and a promoter could be modeled using a repressive Hill function:

$$r_{\text{initiation}}(R)=\frac{1}{1+{\left(\frac{R}{K}\right)}^{n}}\cdot e^{-E_{\text{sc}}∕(k_{B}T)}$$

More generally, we can use stochastic formulations of other regulatory mechanisms and test how these mechanisms behave in concert with supercoiling-mediated feedback.

#### Boundary conditions

Key to our simulations is calculating the supercoiling density across the domain using Equation 2. For simulations using linear boundary conditions, we use the left and right edges as boundaries, assigning excess twist *φ* = 0 at both boundary locations. Then, the supercoiling density can be defined between every polymerase. The location of the boundary conditions for the simulations is described in Table S3.

For simulations using circular boundary conditions, we must define how generated supercoiling “wraps around” the edges of the simulation. To do this, we choose an arbitrary origin, and order polymerases based on their (clockwise) position from the origin. In Table S3, the first boundary location is used as this origin location and the second boundary location is the length of the circle, relative to the origin. As in the linear case, when there are zero polymerases loaded, the supercoiling density is uniformly 0. In addition, for circular boundary conditions, the supercoiling density is *also* uniformly 0 when a single polymerase is present; when assuming fast supercoiling relaxation, a single polymerase on a circle can never accumulate negative or positive supercoiling.

For two or more polymerases, we create a list of excess twists, duplicating the endpoints as *φ*_*n*_, *φ*_1_, ⋯, *φ*_*n*_, *φ*_1_. We additionally project the locations of the wrapped polymerases past the origin (e.g., the position of the wrap-around *φ*_*n*_ is placed at *z*_total length_ – *z_n_*), then compute the supercoiling density as in the linear case.

#### Burst threshold choice

We calculated burst size and inter-burst time by using a burst threshold. From polymerase initiation times, we calculate the time between successive polymerase additions. Intra-addition times greater than the burst threshold form the boundaries between different expression bursts. We define the burst size to be the number of polymerases included in a burst, and the inter-burst time to be those intra-polymerase-addition times greater than the burst threshold. For the main text, we used a burst threshold of 30 s; specifically, this means that bursts ended if 30 s passed without a new polymerase being added.

In Figures S1D and S1E, we reanalyze the data presented in Figures 4C and 4D for different burst thresholds. We find that using a burst threshold of twenty or sixty seconds does not significantly affect the qualitative results observed. However, using a ten second burst threshold does dramatically shift the resulting burst size and inter-burst time distributions, with the inter-burst time distribution becoming concentrated around ten seconds. This indicates that a ten-second burst time is too short and incorrectly separates bursts.

#### Toggle-switch parameter selection

The half-max value *K*, the mRNA count at which the promoter activity is half that of *r*_0_, is chosen here to approximately match the mean steady-state expression of the steady states. The mean steady-state value is identified using simulations where only one of the toggle switch genes is enabled; this allows us to directly account for the influence of supercoiling-mediated feedback on the steady state mRNA concentration. With this choice of *K*, we ensure that the toggle switch operates in the regime of maximum sensitivity (e.g., the stable basin steady-state value is in the middle of the sigmoidal repression curve).

In Figure 5E, we tune the mRNA degradation rate, which directly impacts the mean steady-state value. If the mRNA degradation rate is doubled, we expect that the mean steady-state value should decrease to half its original value. To compare between these otherwise disparate conditions, we scaled the *K* value alongside the mRNA degradation rate, dividing by the fold increase in the mRNA degradation rate.

### QUANTIFICATION AND STATISTICAL ANALYSIS

#### ODE and stochastic simulation

The core ordinary differential equations were simulated using a Tsitouras’s explicit Runge-Kutte 4-5 order method (Tsitouras, 2011). Normally, one implements stochastic simulations using a time-jumping method such as Gillespie’s method. However, because the propensity of our stochastic events changes *continuously* with the continuous simulation, we need a stochastic solver that can be applied within the continuous integrator loop. Here, we used DifferentialEquations.jl, a performant Julia package that allows layered differential and stochastic equations (Rackauckas and Nie, 2017).

#### Summarizing stochastic simulations

Stochastic simulations inherently sample from an underlying population distribution. As single simulations may not adequately represent the population behavior, we simulated ensembles of simulations and chose an ensemble size that was large enough to show the desired population behavior. These simulations then were post-processed, by either taking the average over each ensemble or by directly showing population distributions, summarized by a kernel density estimate (smoothed histograms provided by the Python package seaborn). The ensemble size for each data plot is summarized in Table S1.

#### Computing cross-correlation

We computed the cross-correlation between the gene outputs following induction (Figure 3C), normalized by the geometric mean of the auto-correlation of these outputs. Strong negative or positive peaks evenly spaced around *τ* = 0 is a hallmark of periodic behavior, with the time offset of the peak encoding the phase offset between the signals. For two signals *f*(*t*) and *g*(*t*), the normalized cross-correlation at a certain time offset *τ* is bounded between ± 1 and can be thought of as the correlation coefficient between the unshifted version of one of the signals (*f*(*t*)) and the other signal shifted in the time axis by *τ*(*g*(*t*+*τ*)). Mathematically, we use:

(Equation 10) $$\text{cross}-\text{correlation}(\tau )=\frac{\left(f⋆g)(\tau \right)}{\sqrt{\left(f⋆f)(0)\cdot (g⋆g)(0\right)}}$$

for

(Equation 11) $$\left(f⋆g)(\tau )=\sum_{t}(f(t)-\langle f(t)\rangle )(g(t+\tau )-\langle g(t)\rangle \right)$$

In fact, for *τ* = 0, the normalized cross-correlation of the two signals is exactly the Pearson correlation coefficient. The shape of the cross-correlation curve can also reveal periodic and other time-dependent correlative behaviors.

## Supplementary Material

1

2

3

4

5

## Figures and Tables

**Table:** KEY RESOURCES TABLE

REAGENT or RESOURCE	SOURCE	IDENTIFIER
Deposited data		
Simulation datasets	This work	https://doi.org/10.5281/zenodo.7041641
Software and algorithms		
Adobe Illustrator CC	Adobe Systems	https://www.adobe.com
Julia 1.6.1		https://julialang.org
DifferentialEquations.jl 6.19.0	Rackauckas and Nie, 2017	https://diffeq.sciml.ai/stable
Python 3		https://www.python.org
Simulation and figure-generation code	This work	https://doi.org/10.5281/zenodo.7054394

## Highlights

- Supercoiling dynamics confer rapid, tunable coupling between adjacent genes
- Syntax—the relative order and orientation of genes—alters expression levels
- Supercoiling-dependent feedback tunes transcriptional variance and bursting
- Supercoiling coordinates the dynamics of transcriptional networks and gene circuits
