# Supplementary material for: Supercoiling-mediated feedback rapidly couples and tunes transcription
Source: Cell Rep. Author manuscript; Available in PMC 2022 Nov 1. (PMC9624111; doi:10.1016/j.celrep.2022.111492)
Supplement: 1 [file NIHMS1843689-supplement-1.pdf]

Cell Reports, Volume *41*

Supplemental information

Supercoiling-mediated feedback rapidly couples and tunes transcription

Christopher P. Johnstone and Kate E. Galloway

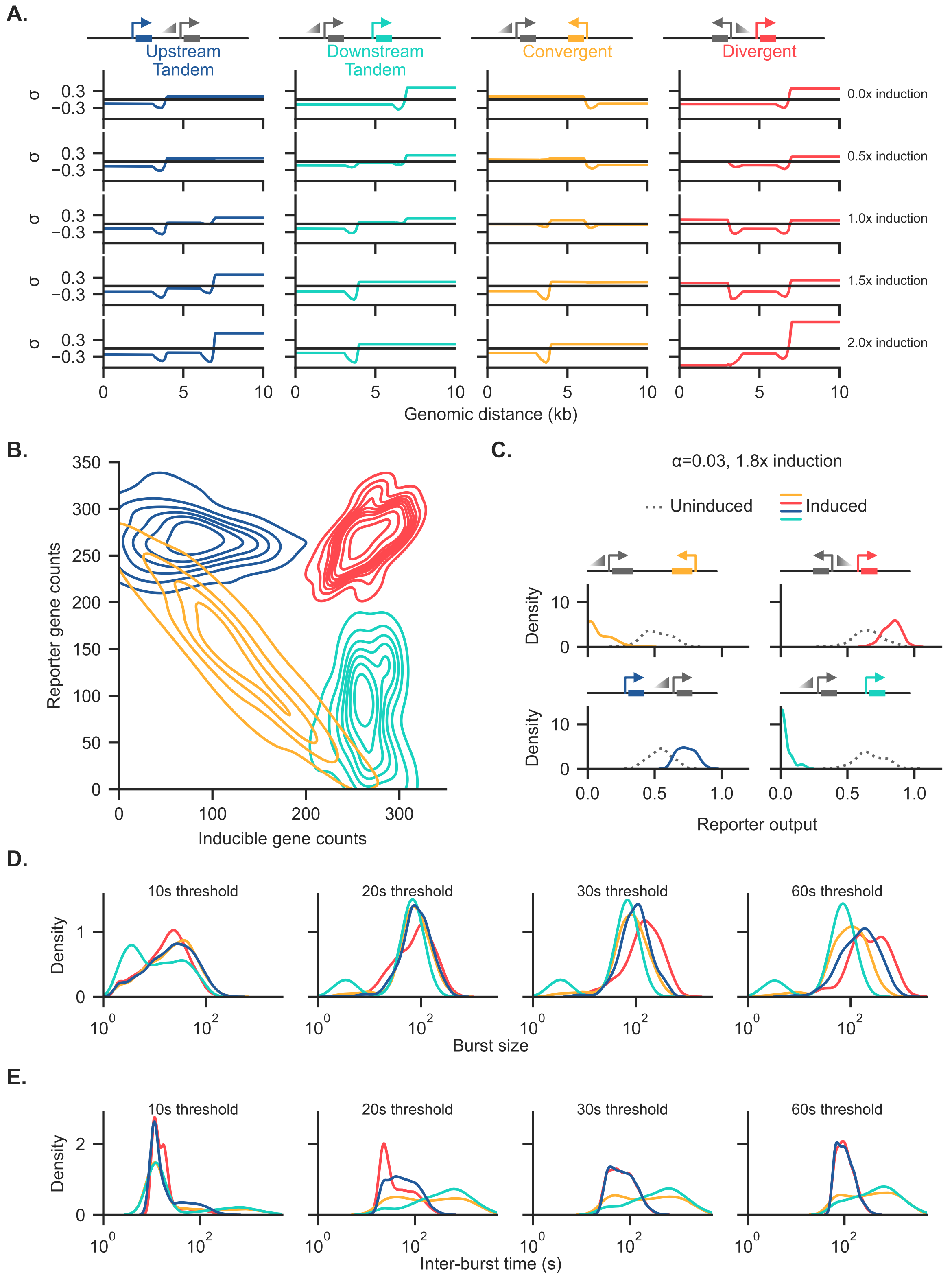

A.
Upstream Tandem
Downstream Tandem
Convergent
Divergent
σ
0.3
−0.3
0.0x induction
0.5x induction
1.0x induction
1.5x induction
2.0x induction
0
5
10
Genomic distance (kb)
B.
Reporter gene counts
Inducible gene counts
C.
α=0.03, 1.8x induction
Uninduced
Induced
Density
Reporter output
D.
10s threshold
20s threshold
30s threshold
60s threshold
Density
Burst size
E.
Inter-burst time (s)

Figure S1: Related to figs. 2 to 4. a) Supercoiling density profiles are plotted for a wide range of adjacent gene induction levels across the four syntaxes considered. At low adjacent induction of the adjacent gene, positive and negative supercoiling mainly accumulates upstream and downstream, respectively, of the reporter gene. At 1-fold (equal base rates) induction, the supercoiling distributions are nearly symmetric in the convergent and divergent syntaxes. At high adjacent induction, the adjacent gene expression dominates the generated supercoiling profiles. b) The distribution of inducible and reporter mRNA counts are shown for the simulation ensembles presented in fig. 3. The different initial starting condition does not affect the final distribution state when compared to fig. 2d, indicating that the final state reached is independent of initial state. c)The ensemble distribution of the four linear circuit syntaxes is shown for $\alpha = 0.03$ and high induction of the adjacent gene (1.8 fold). d),e) Burst size and inter-burst time distributions are shown at equal-induction as a function of threshold time and circuit syntax. At the lowest threshold time, the burst size and inter-burst time distributions qualitatively differ, with only a small percentage of bursts having an inter-burst time larger than 20 seconds. This lack of larger inter-burst times suggests that a burst threshold of at least 20 seconds gives useful predictions.

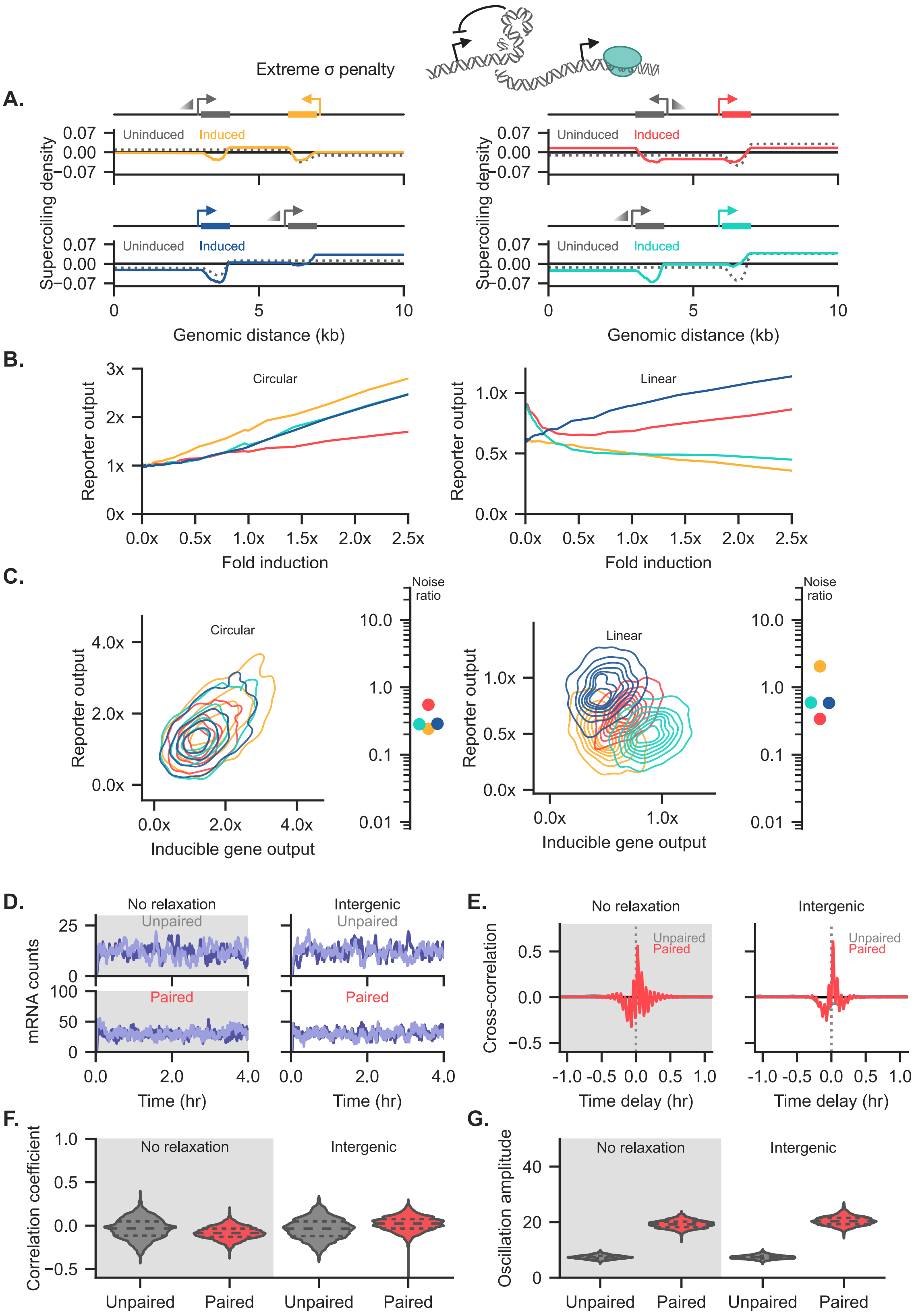
Extreme σ penalty
A.
Uninduced
Induced
Supercoiling density
0.07
0.00
−0.07
0
5
10
Genomic distance (kb)
B.
Circular
Linear
Reporter output
3x
2x
1x
0x
1.0x
0.5x
0.0x
0.0x
0.5x
1.0x
1.5x
2.0x
2.5x
Fold induction
C.
Circular
Linear
Reporter output
4.0x
2.0x
0.0x
Inducible gene output
Noise ratio
10.0
1.0
0.1
0.01
D.
No relaxation
Intergenic
Unpaired
Paired
mRNA counts
25
0
100
50
0.0
2.0
4.0
Time (hr)
E.
No relaxation
Intergenic
Unpaired
Paired
Cross-correlation
0.5
0.0
−0.5
-1.0
-0.5
0.0
0.5
1.0
Time delay (hr)
F.
Correlation coefficient
1.0
0.5
0.0
−0.5
No relaxation
Intergenic
Unpaired
Paired
G.
Oscillation amplitude
40
20
0
No relaxation
Intergenic
Unpaired
Paired

Figure S2: Related to Figures 2, 4 and 6. Simulation of an RNAP binding penalty at highly positive and negative supercoiling densities reduces syntax-specific differences and impedes transcriptional coupling between *her1*and *her7*. a) The average supercoiling density for both the induced and induced case is shown. The average supercoiling density is smaller than observed in figs. 3b and S3a due to the inhibition of polymerase initiation at extreme values of supercoiling density. b) Reporter output as a function of adjacent gene induction and circuit syntax is shown for both circular and linear boundary conditions. c) The population distribution is shown as a function of boundary condition and circuit syntax for the equally-induced case. The noise ratio of each is defined as the intrinsic noise (off-diagonal component) divided by the extrinsic noise (on-diagonal component). d) Four example simulation traces of the zebrafish segmentation gene network are shown, for two topoisomerase conditions and the gene-unpaired and gene-paired orientations. No clear periodicity is observed. e) The ensemble cross-correlation for the zebrafish segmentation gene network is shown. In this restricted polymerase initiation case, strong periodicity is not observed. f) The ensemble distribution of the correlation coefficient between *her1* and *her7* is shown. Restricting polymerase initiation eliminates *her1*/*her7* correlation, even in the gene-paired case. g) The ensemble oscillation amplitude is shown for each topoisomerase and gene pairing condition.

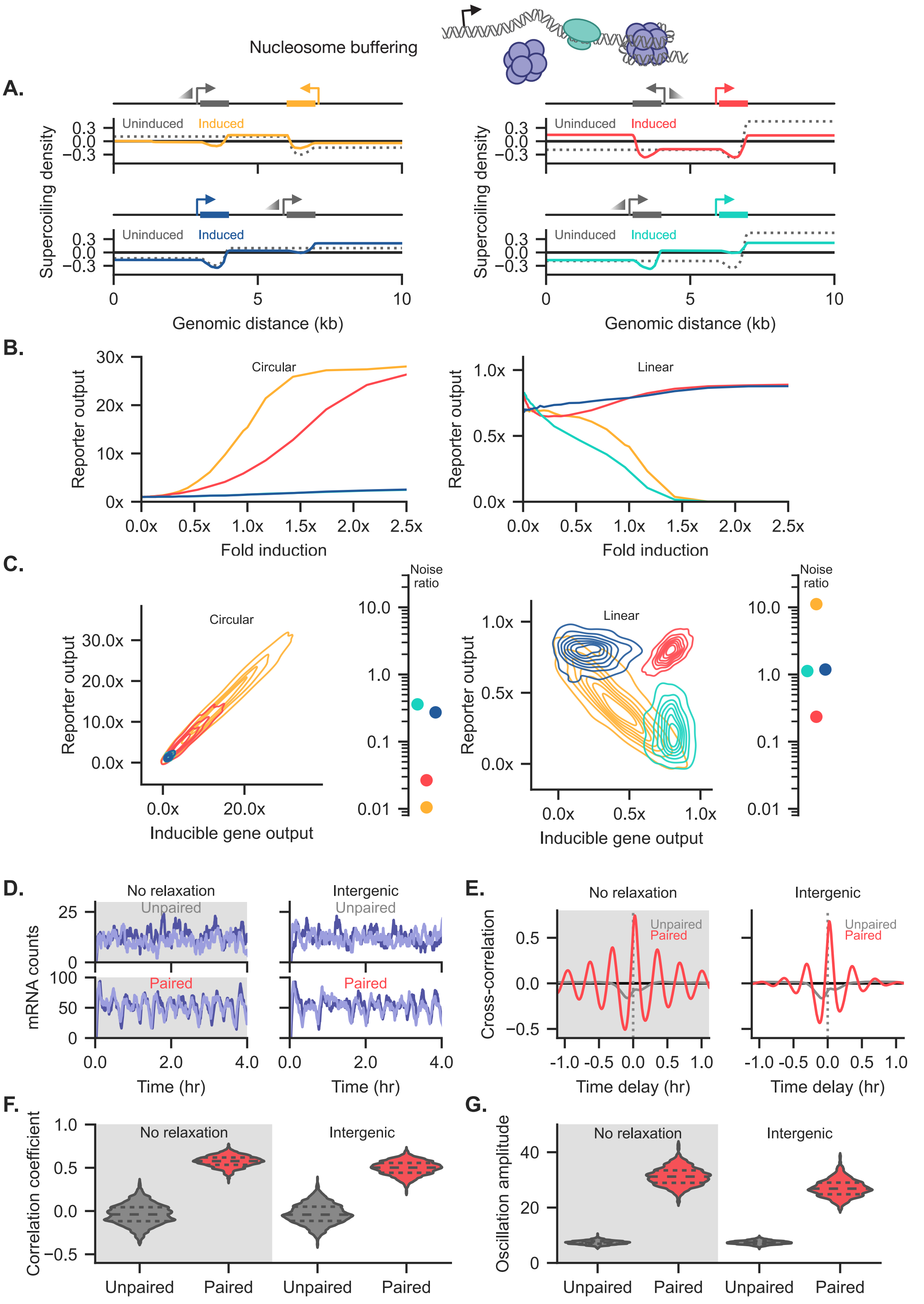

Nucleosome buffering
A.
Uninduced
Induced
Supercoiling density
0.3
0.0
−0.3
0
5
10
Genomic distance (kb)
B.
Circular
Reporter output
30x
20x
10x
0x
0.0x
0.5x
1.0x
1.5x
2.0x
2.5x
Fold induction
Linear
1.0x
0.5x
0.0x
C.
Circular
Reporter output
30.0x
20.0x
10.0x
0.0x
20.0x
Inducible gene output
Noise ratio
10.0
1.0
0.1
0.01
Linear
1.0x
0.5x
0.0x
D.
No relaxation
Unpaired
Paired
mRNA counts
25
0
100
50
Intergenic
Time (hr)
0.0
2.0
4.0
E.
No relaxation
Intergenic
Cross-correlation
Unpaired
Paired
0.5
0.0
−0.5
-1.0
-0.5
0.0
0.5
1.0
Time delay (hr)
F.
Correlation coefficient
1.0
0.5
0.0
−0.5
No relaxation
Intergenic
Unpaired
Paired
G.
Oscillation amplitude
40
20
0

Figure S3: Related to Figures 2, 4 and 6. Nucleosome buffering of positive supercoiling displays weak effects on the expression profiles of two-gene systems at steady state and on the *her1-her7* somite network. a) The average supercoiling density for both the induced and induced case is shown. b) Reporter output as a function of adjacent gene induction and circuit syntax is shown for both circular and linear boundary conditions. c) The population distribution is shown as a function of boundary condition and circuit syntax for the equally-induced case. The noise ratio of each is defined as the intrinsic noise (off-diagonal component) divided by the extrinsic noise (on-diagonal component). d) Four example simulation traces of the zebrafish segmentation gene network are shown, for two topoisomerase conditions and the gene-unpaired and gene-paired orientations. e) The ensemble cross-correlation for the zebrafish segmentation gene network with nucleosome buffering is shown. Robust periodicity in the gene-paired case remains in the presence of nucleosomes. f) The ensemble distribution of the correlation coefficient between *her1* and *her7* is shown. In the presence of nucleosomes, the gene-paired case has a significantly increased correlation coefficient. g) The ensemble oscillation amplitude is shown for each topoisomerase and gene pairing condition.

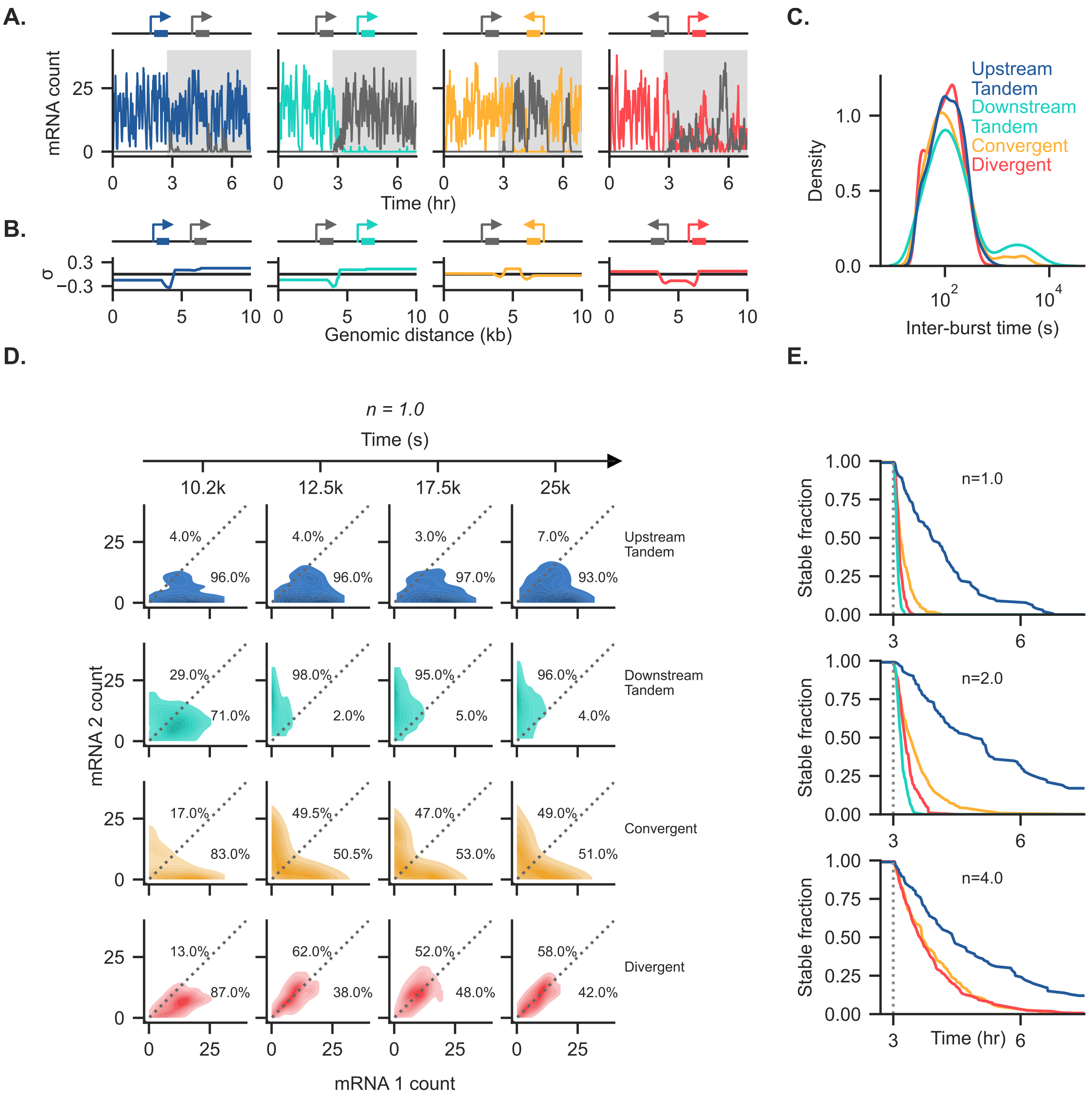

Figure S4: Related to Figure 5. a) Four example simulation runs for the toggle switches are placed in the four orientations. b) The mean ensemble supercoiling density is plotted for the four different circuit syntaxes. c) The distribution of inter-burst times for the four different toggle switch architectures is shown. d) The ensemble mRNA count distributions are shown as a function of syntax at four selected time points for $n = 1.0$. Even as early as 200 seconds after induction of the second gene, we see that the tandem-downstream and divergent syntaxes are quickly approaching their equilibrium ensemble distributions. e) The basin stability for the four toggle-switch architectures is shown explicitly as a function of Hill coefficient, n.

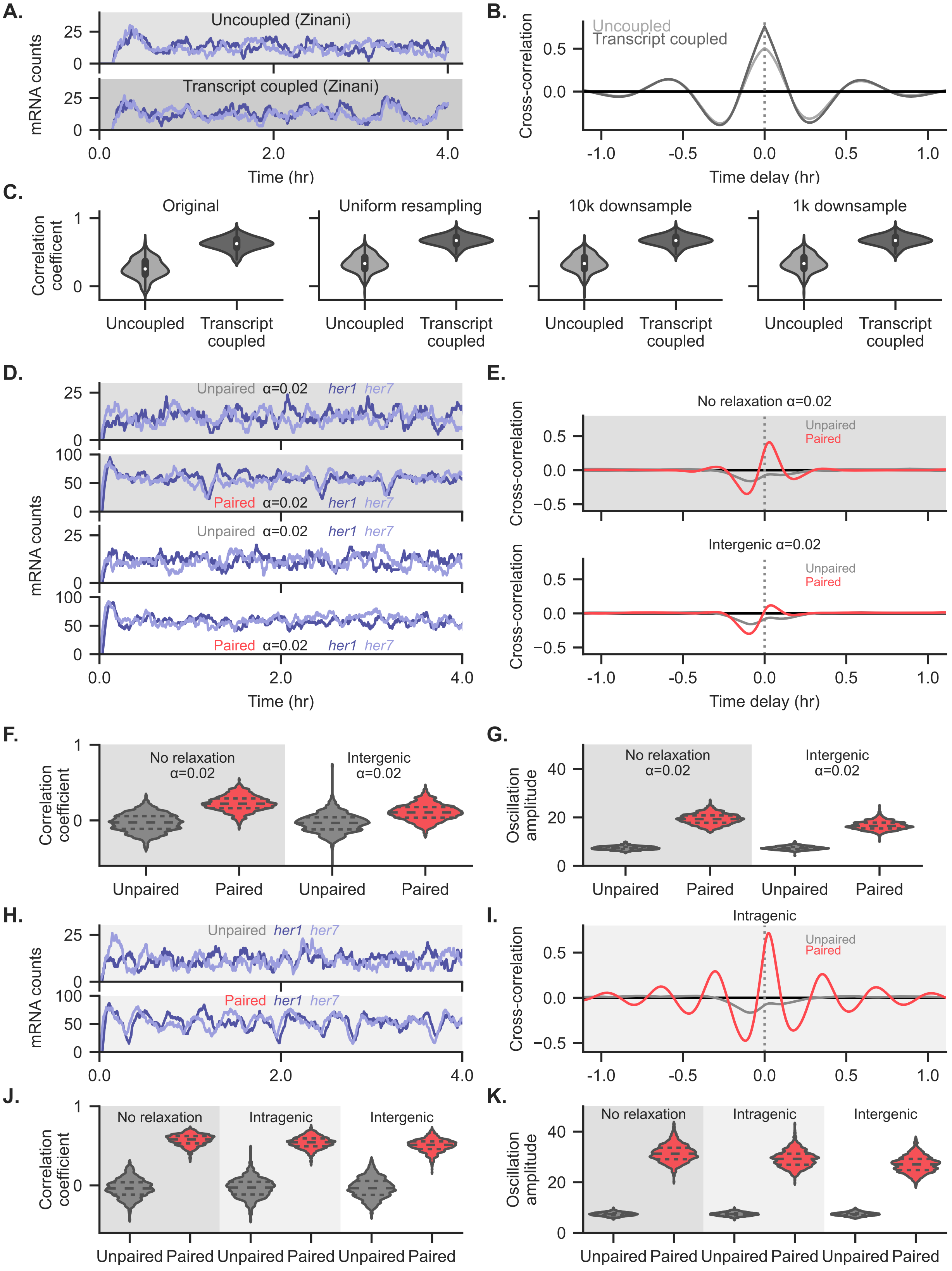
A.
Uncoupled (Zinani)
Transcript coupled (Zinani)
mRNA counts
Time (hr)
B.
Uncoupled
Transcript coupled
Cross-correlation
Time delay (hr)
C.
Original
Uniform resampling
10k downsample
1k downsample
Correlation coefficent
Uncoupled
Transcript coupled
D.
Unpaired α=0.02 her1 her7
Paired α=0.02 her1 her7
mRNA counts
Time (hr)
E.
No relaxation α=0.02
Intergenic α=0.02
Unpaired
Paired
Cross-correlation
Time delay (hr)
F.
No relaxation α=0.02
Intergenic α=0.02
Correlation coefficient
Unpaired
Paired
G.
Oscillation amplitude
H.
Unpaired her1 her7
Paired her1 her7
mRNA counts
I.
Intragenic
J.
No relaxation
Intragenic
Intergenic
K.

Figure S5: Related to Figure 6. a) Using the authors' MATLAB implementation of the model in Zinani et al. (2021), we simulated ensembles of 10,000 runs in order to compare to our simulation work. Unlike our implementation, which does not contain extra delays not due to polymerase motion and stalling, the original model uses a fixed mRNA production delay time of 9 minutes and a fixed protein production delay time of 1 minute. Example runs of the original model are shown. The transcript-coupled case shows correlated but relatively aperiodic behavior. b) The ensemble cross-correlation shows that the uncoupled and transcript coupled cases in the original model show very similar correlation structures, showing that both have similar amounts of periodicity in the original model. c) Using Zinani's original MATLAB code, we computed the correlation coefficient distributions using both their original sampling method (far-left) and with the dataset resampled uniformly in time. The qualitative results between these two sampling methods remain the same, with the original sampling method slightly under-reporting *her1*-*her7* correlation due to its reliance on Gillespie-sampled timepoints. We further found that down-sampling the uniformly-sampled dataset did not noticeably change the correlation distributions. d)-f) Re-simulation of the *her1*-*her7* system presented in fig. 6 is shown for $\alpha = 0.02$, a choice that occurs in the first regime of supercoiling-dependent initiation behavior. Strikingly, the strong correlated behavior seen in fig. 6 is absent at this choice of α, suggesting that supercoiling-dependent initiation is an important phenomena in the synchronization of *her1* and *her7* activity. d) The number of *her1* and *her7* mRNAs are shown in the two different simulation contexts. e) For $\alpha = 0.02$, the biophysically-coupled case no longer shows strong periodic behavior in the cross-correlation plots, instead showing similar asymmetric cross-correlation behavior. f) In contrast to fig. 6e, for $\alpha = 0.02$, the gene paired system now shows a weaker correlated expression. g) The mean oscillation amplitude is shown for the gene unpaired and gene paired cases with intragenic topoisomerase relaxation. The gene paired case still shows an increase in average oscillation amplitude. h) Example mRNA traces are shown for the zebrafish segmentation network with intragenic topoisomerase relaxation. i) The cross correlation of clock gene expression is shown for intragenic topoisomerase relaxation. j) The correlation coefficient between *her1 her7* is shown, compared across the three different topoisomerase activity conditions. k) The size of oscillations is compared across the three different topoisomerase activity conditions.

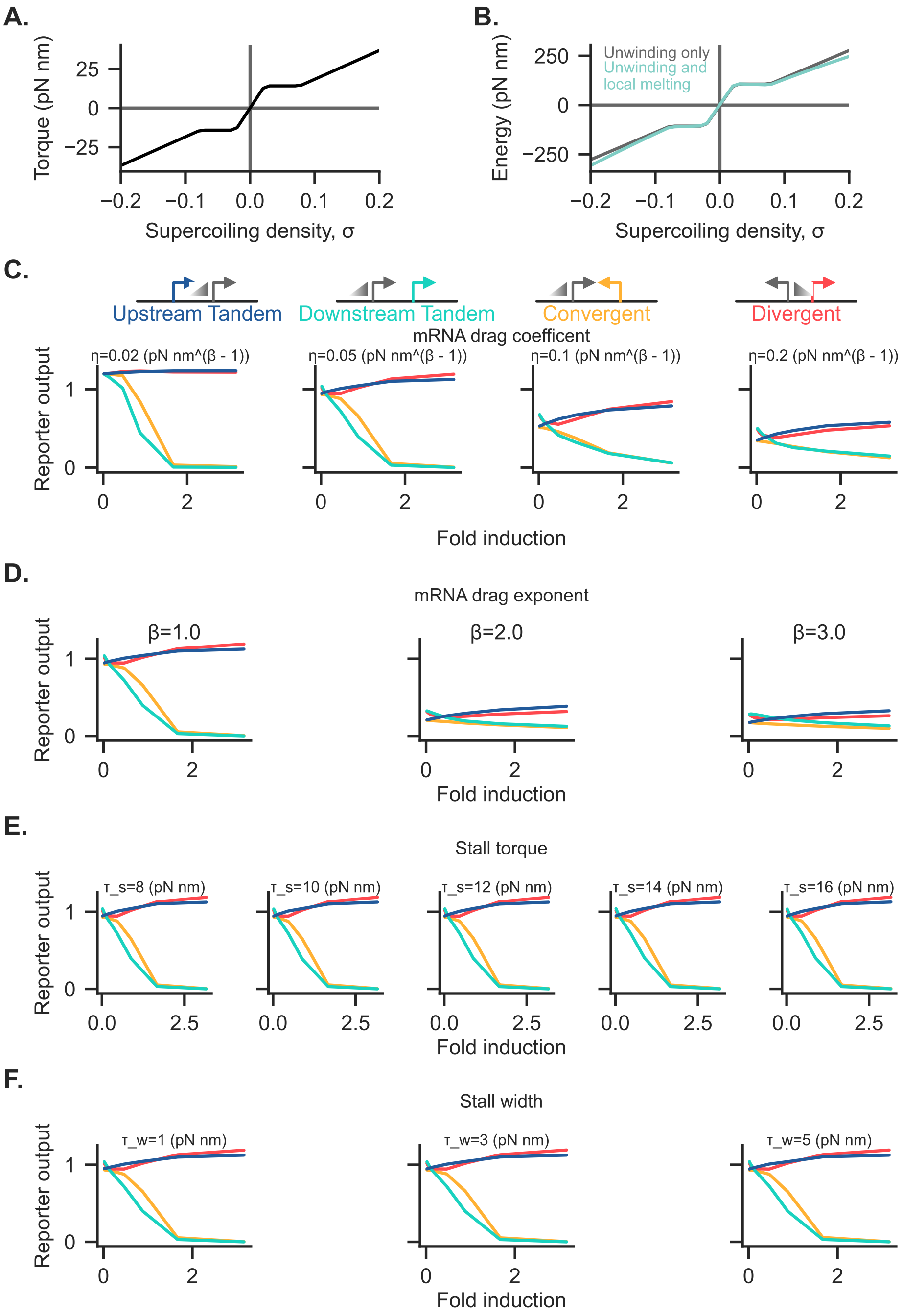
A.
Torque (pN nm)
Supercoiling density, σ
B.
Energy (pN nm)
Unwinding only
Unwinding and local melting
Supercoiling density, σ
C.
Upstream Tandem
Downstream Tandem
Convergent
Divergent
mRNA drag coefficent
η=0.02 (pN nm^(β - 1))
η=0.05 (pN nm^(β - 1))
η=0.1 (pN nm^(β - 1))
η=0.2 (pN nm^(β - 1))
Reporter output
Fold induction
D.
mRNA drag exponent
β=1.0
β=2.0
β=3.0
Reporter output
Fold induction
E.
Stall torque
τ_s=8 (pN nm)
τ_s=10 (pN nm)
τ_s=12 (pN nm)
τ_s=14 (pN nm)
τ_s=16 (pN nm)
Reporter output
Fold induction
F.
Stall width
τ_w=1 (pN nm)
τ_w=3 (pN nm)
τ_w=5 (pN nm)
Reporter output
Fold induction

Figure S6: Related to STAR Methods. Reporter output of two-gene circuits with linear boundary conditions (as in fig. 2) demonstrates similar qualitative behavior when underlying model parameters are changed. a) The torque response predicted by Marko's model (eq. (S1)) exhibits phase behavior, with a transition occurring between a over- and under-twisted phase to a largely plectonemic phase. During the transition, the torque response is constant. b) For the supercoiling-dependent initiation model, the energy that it takes to introduce supercoiling upstream and downstream by unwinding the DNA dominates the energy expression. Including a term representing the energy of local melting of the double helix at the location of the polymerase only gives a minor correction. c) Varying the nascent mRNA drag coefficient from the chosen value $\eta = 0.05$ rescales but does not dramatically change reporter output behavior. Larger drag coefficients increase generated supercoiling by introducing additional torque into the system. d) Varying the exponent on the nascent mRNA length in the drag coefficient from the choice of linear drag $\beta = 1.0$ also rescales but does not dramatically change reporter output behavior. Choices of $\beta > 1.0$ tends to increase generated supercoiling due to longer nascent mRNAs having an increased drag profile. e) Varying the RNAP stall torque from the chosen $\tau_s = 12$ pN nm has a minor effect on reporter output. f) Varying the width of the stalling function from the chosen $\tau_w = 3$ pN nm has no noticeable effect on reporter output.

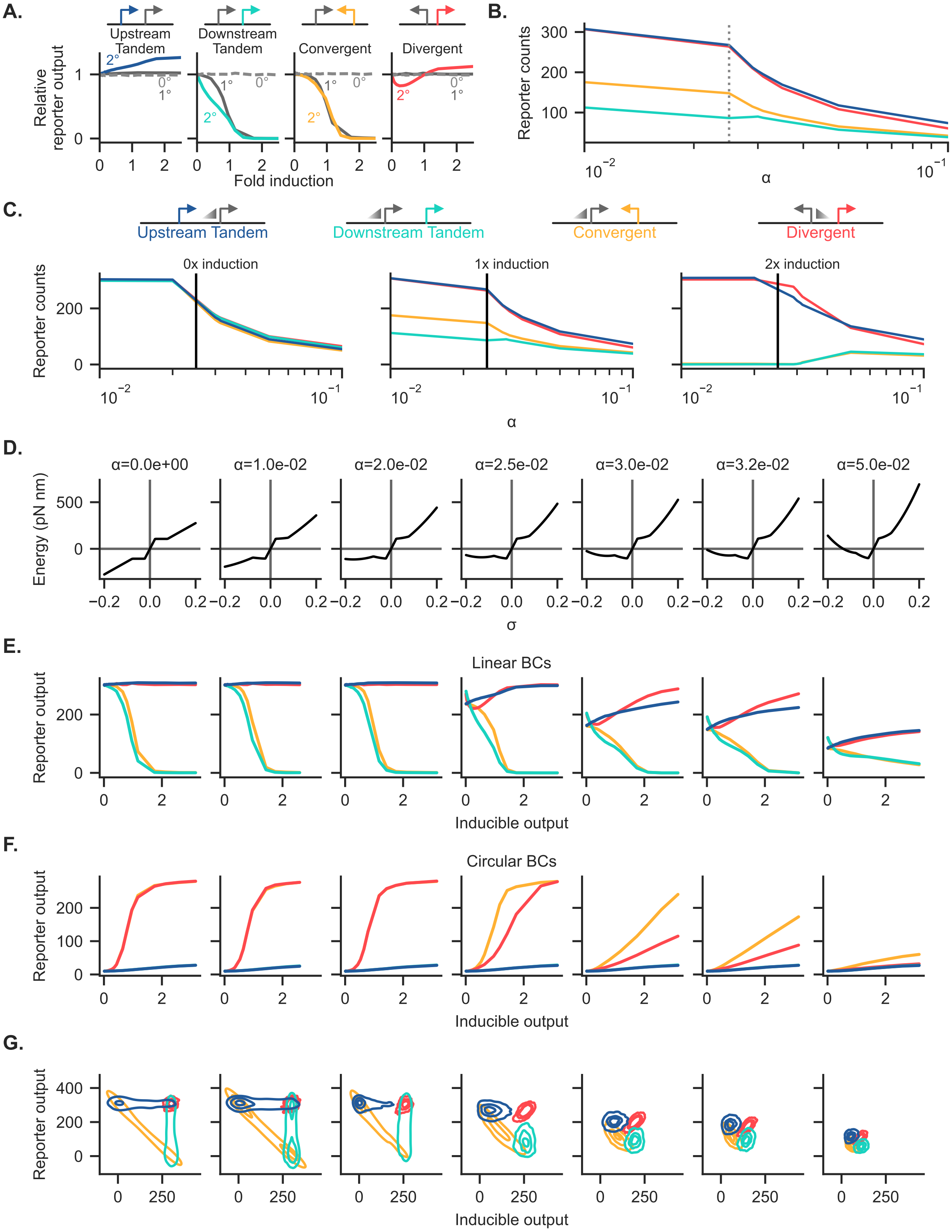

A.
Upstream Tandem
Downstream Tandem
Convergent
Divergent
Relative reporter output
Fold induction
B.
Reporter counts
α
C.
Upstream Tandem
Downstream Tandem
Convergent
Divergent
0x induction
1x induction
2x induction
Reporter counts
α
D.
α=0.0e+00
α=1.0e-02
α=2.0e-02
α=2.5e-02
α=3.0e-02
α=3.2e-02
α=5.0e-02
Energy (pN nm)
σ
E.
Linear BCs
Reporter output
Inducible output
F.
Circular BCs
Reporter output
Inducible output
G.
Reporter output
Inducible output

Figure S7: Related to STAR Methods. As the value of the quadratic energy correction coefficient α varies, two regimes of supercoiling-dependent behaviors emerge. a) Reporter output is shown, normalized to the zero-fold induction case for the four tested syntaxes for the three different polymerase initiation models. b) For the second-order model, we chose the value of α that defined the transition between regimes. c) For $\alpha \leq 0.02$, supercoiling-dependent initiation appears to dominate, leading to both the maintenance of reporter output in the linear divergent case and near-equality of the convergent and divergent cases with circular boundary conditions. For $\alpha \geq 0.025$, emergent non-monotonic behavior appears, leading to complex behaviors with linear boundary conditions and a separation between the response of the convergent and divergent syntaxes with circular boundary conditions. d) The additional polymerase binding energy as a function of supercoiling density is plotted for various values of α. e) f) Reporter behavior of the four syntaxes under linear and circular boundary conditions are shown, in comparison to fig. 2b. g) The overall distribution of the four syntaxes as a function of alpha is shown, in comparison to fig. 2d.

Plot description	n	Figures
Each point comprising the plotted curves is an average over n simulations.	750	figs. S2b and S3b
	2000	figs. 2b, S6c to S6f, S7a, S7c, S7e and S7f
	5000	fig. 2f
	10000	figs. 5c, 5e and S4e
Each line shows the average supercoiling density profile over an ensemble of n simulations.	100	figs. 2e, S1a and S4b
	250	figs. 3b, S2a and S3a
The standard deviation of gene output for an ensemble of n simulations is shown as a function of time.	250	fig. 3f
The average cross-correlation over an ensemble of n simulations is plotted.	250	fig. 3d
	2000	figs. 6d, S2e, S3e, S5e and S5i
	10000	fig. S5b
Distribution profiles are shown over an ensemble of n simulations. One-dimensional distributions are shown as a kernel density estimate (smoothed histogram) or violin plot. Two-dimensional distributions are shown as filled or unfilled isocurves.	100	fig. 4c (base model),fig. 4d (base model), figs. S1b, S1d, S1e and S4c
	250	figs. 3e and S1c,fig. 4c (model perturbations), fig. 4d (model perturbations)
	750	figs. S2c and S3c
	2000	figs. 2c, 2d, 6e, 6f, S2f, S2g, S3f, S3g, S5f, S5g, S5j, S5k and S7g
	10000	figs. 5b, 5d, S4d and S5c
An example simulation was randomly selected from an ensemble of n simulations and plotted.	250	fig. 3a
	2000	figs. 6c, S2d, S3d, S5d and S5h
	10000	figs. S4a and S5a

Table S1: Key ensemble size and interpretive description of each plot. Related to STAR Methods.

Drag coefficient η (pN nm$^{\beta-1}$)	Drag exponent β	Stall torque τ_s (pN nm)	Stall width τ_w (pN nm)	Figures
1/20	1	12	3	All except figs. S6c to S6f
1/50$\longleftrightarrow$ 1/5	1	12	3	Figure S6c
1/20	1$\longleftrightarrow$3	12	3	Figure S6d
1/20	1	8$\longleftrightarrow$16	3	Figure S6e
1/20	1	12	1$\longleftrightarrow$5	Figure S6f

Table S2: Key dynamic constants for all simulations. Related to STAR Methods.

Type	Boundary locations	Reporter gene	Inducible gene	Figures
Upstream tandem	0, 10000	3000$\rightarrow$4000	6000$\rightarrow$7000	Figures 2b to 2e, 3, 4, S1a to S1e, S2a to S2c, S3a to S3c, S6c to S6f, S7 and S7a
Downstream tandem	0, 10000	6000$\rightarrow$7000	3000$\rightarrow$4000	
Convergent	0, 10000	7000$\rightarrow$6000	3000$\rightarrow$4000	
Divergent	0, 10000	6000$\rightarrow$7000	4000$\rightarrow$3000	
Type	Boundary locations	Reporter gene	Inducible gene	Figures
Upstream tandem	0, $8000 + \Delta x$	3000$\rightarrow$4000	$4000 + \Delta x \rightarrow 5000 + \Delta x$	Figure 2f
Downstream tandem	0, $8000 + \Delta x$	$4000 + \Delta x \rightarrow 5000 + \Delta x$	3000$\rightarrow$4000	
Convergent	0, $8000 + \Delta x$	$5000 + \Delta x \rightarrow 4000 + \Delta x$	3000$\rightarrow$4000	
Divergent	0, $8000 + \Delta x$	$4000 + \Delta x \rightarrow 5000 + \Delta x$	4000$\rightarrow$3000	
Type	Boundary locations	Gene A	Gene B	Figures
Upstream tandem	0, 10000	3500$\rightarrow$4500	5500$\rightarrow$6500	Figures 5 and S4c to S4e
Downstream tandem	0, 10000	5500$\rightarrow$6500	3500$\rightarrow$4500	
Convergent	0, 10000	3500$\rightarrow$4500	6500$\rightarrow$5500	
Divergent	0, 10000	4500$\rightarrow$3500	5500$\rightarrow$6500	
Type	Boundary locations	*her1*	*her7*	Figures
Gene-unpaired	0, 10^9 (free ends)	6405$\rightarrow$6017	$(10^9 - 9864) \rightarrow (10^9 - 8549)$	Figures 6, S2d to S2g, S3d to S3g and S5d to S5k
Gene-paired	0, 34393	12422$\rightarrow$6017	24529$\rightarrow$25844	

Table S3: Simulated circuit locations and distances. Locations are all given in basepairs (with 0.34 nanometers per base pair). For the given genes, the first number given is the simulated promoter location and start of the gene body, and the second number is a simulated termination site. Related to STAR Methods.

Methods S1: Model derivation

Our model builds upon several prior papers and relevant mathematical frameworks. To ensure access and provide definitions needed for implementation of our model, we have aggregated all of these relevant equations and simulation constants together. From this foundation, we discuss how we have extended our model to examine how DNA supercoiling generates coupling between proximal genes.

Literature supercoiling model

Why do we care about supercoiling at all? From statistical mechanics, the thermal energy unit $k_B T$, defined by the product of the Boltzmann constant and the temperature of the system, defines an energy accessible by fluctuations; useful chemical reactions and interactions often occur above this fluctuation energy. Converting into units relevant to supercoiling at $T = 300$ K, $k_b T = 4.1$ pN nm, Many of the later quantities we will solve for will have magnitude around 10-100 pN nm—well above the thermal energy—-so can determining system behavior.

When we derived the supercoiling density, σ, in eq. (2), we defined ϕ to be the local excess twist. While this term is most easily identified with the local DNA excess twist, it is truly a linking number constraint that takes into account both twist, local stretching of bonds that causes more or fewer complete rotations to occur over a set distance, and writhe, where mesoscopic plectonemes store supercoiling. These two forms of supercoiling are physically interconvertable, but are not energetically identical. This energetic difference is accounted for in the underlying statistical mechanical model (fig. S6a).

The statistical mechanics model depends on the following constants:

Variable	Value	Reference
k_b, Boltzmann constant	$0.01381 \frac{\text{pN nm}}{\text{K}}$	-
ω_0, relaxed DNA twist frequency	$1.85 \frac{1}{\text{nm}}$	(Stuart A Sevier and Levine 2018), others
A, bend persistence length	50nm	(Marko 2007)
C, twist persistence length (twisted)	95 ± 10nm	(Marko 2007)
P, twist persistence length (plectonemic)	24 ± 3nm	(Marko 2007)
f, applied force on DNA	1 pN	(Stuart A Sevier and Levine 2018)

Table S4: Key values for the statistical mechanics model

Especially key measurable constants are the bend persistence length A, the twist persistence length of stretched DNA C, and the twist persistence length of the plectonemic state P. Because plectonemes convert twist into writhe, we expect $P < C$.

Marko defines the following rescaled versions of P and C, p, c:

$$c = k_B T C \omega_0^2 \qquad p = k_B T P \omega_0^2$$

which in turn can be used to define helper constants g, c_s:

$$g = f - \sqrt{\frac{k_B T f}{A}} \qquad c_s = c\left[1 - \frac{C}{4A}\sqrt{\frac{k_B T}{Af}}\right]$$

With these defined, Marko's statistical-mechanics model calculates the torque as a function of supercoiling density (fig. S6a) (Marko 2007):

$$\tau(\sigma) = \begin{cases} \tau_S \sigma & |\sigma| < |\sigma_s| \\ \tau_0 \text{sgn}(\sigma) & |\sigma_s| \le |\sigma| \le |\sigma_p| \\ \tau_P \sigma & |\sigma_p| \le |\sigma| \end{cases} \tag{S1}$$

for critical supercoiling values σ_s, σ_p separating the phase regimes:

$$|\sigma_s| = \frac{1}{c_s}\sqrt{\frac{2pg}{1-\frac{p}{c_s}}} \qquad |\sigma_p| = \frac{1}{p}\sqrt{\frac{2pg}{1-\frac{p}{c_s}}} \tag{S2}$$

with corresponding critical torque values τ_S, τ_P:

$$\tau_S = \frac{c_s}{\omega_0} \qquad \tau_0 = \sqrt{\frac{2pg}{\omega_0^2\left(1-\frac{p}{c_s}\right)}} \qquad \tau_P = \frac{p}{\omega_0} \tag{S3}$$

Using the torque equation eq. (S1), Marko expands the free energy of the DNA per unit length, S, as a power series in σ:

$$S = \begin{cases} -g + \frac{1}{2}c_s\sigma^2 & |\sigma| < |\sigma_s| \\ \frac{-g}{1-\frac{p}{c_s}} + \sqrt{\frac{2pg}{1-\frac{p}{c_s}}}|\sigma| & |\sigma_s| < |\sigma| < |\sigma_p| \\ \frac{1}{2}p\sigma^2 & |\sigma| > |\sigma_p| \end{cases} \tag{S4}$$

While the cubic term can also be calculated and accounts for asymmetry between under- and over-winding, Marko (2007) argues that this effect is small for realistic physiological conditions.

Literature dynamic model

Following Stuart A Sevier and Levine (2018), we adopt a dynamics model for modeling the motion of polymerases over the DNA. This model depends on the following constants:

Variable	Value	Reference
χ, DNA twist mobility	0.01381 s pN nm	(Stuart A Sevier and Levine 2018)
η, mRNA drag coefficent	1/20 pN	(Stuart A Sevier and Levine 2018)
n, Drag scaling exponent	1	(Stuart A Sevier and Levine 2018)
v_0, Maximum polymerase velocity	$20\frac{\text{nm}}{\text{s}}$	(Stuart A Sevier and Levine 2018)
τ_s, Polymerase stall torque	12 pN nm	(Stuart A Sevier and Levine 2018)
$\Delta\tau_s$, Polymerase stall width	3 pN nm	This work
δ, Polymerase radius	15 nm	(Stuart A Sevier and Levine 2018)

Table S5: Key dynamic model values

Briefly, for each polymerase, this model tracks the position z_i, transcript length x_i, and excess DNA twist ϕ_i of each RNAP. We create a *linking number constraint* by specifying ϕ at any genomic location, which means that each RNAP sets an linking number constraint. We use dynamic equations to model the motion of RNAP against either fixed or free boundary conditions.

As mentioned in the main text, the dynamic equations are:

$$\omega_0\frac{dz_i}{dt} = \frac{d\theta_i}{dt} + \frac{d\phi_i}{dt} \qquad \tau(z_i, \phi_{i-1}, \phi_{i+1}) = \eta x_i^n \frac{d\theta_i}{dt} - \chi\frac{d\phi_i}{dt}$$

By solving the left equation for $\frac{d\theta_i}{dt}$, the polymerase angular velocity, and substituting it into the right equation, we get:

$$\tau(z_i, \phi_{i-1}, \phi_{i+1}) = \omega_0\frac{dz_i}{dt}\eta x_i^n - \eta x_i^n\frac{d\phi_i}{dt} - \chi\frac{d\phi_i}{dt}$$

Solving for $\frac{d\phi_i}{dt}$, we get a dynamic equation for the differential linking number constraint:

$$\frac{d\phi_i}{dt} = \omega_0 \frac{dz_i}{dt} \frac{\eta x_i^n}{\chi + \eta x_i^n} - \frac{\tau(z_i, \phi_{i-1}, \phi_{i+1})}{\chi + \eta x_i^n} \tag{S5}$$

To model polymerase stalling, we use a parameterized function discussed in eq. (5) that explicitly depends on a stall torque and a torque width τ_w over which stalling begins to occur.

Equations B.2 and 5 along with the definition of linear velocity $\frac{dx_i}{dt} = \frac{dz_i}{dt}$ together represent a complete coupled set of ODEs that can be simulated, as long as the following constants are defined:

Model extension: supercoiling-dependent initiation

Reflecting on the desired properties of a model based on literature observations, we extended the models from Sevier and Marko to account for supercoiling-dependent initiation. The rest of this appendix details this process, in addition to the initial model formulation and useful simplifications.

Literature observations and modeling goals

A naked DNA bead experiment (Revyakin, Ebright, and Strick (2004)) found that positive and negative supercoiling affected RNAP polymerase binding rates, with negative supercoiling encouraging binding and positive supercoiling discouraging binding. When a polymerase bound, approximately 1.2 turns of DNA unwound (13 base pairs, or 4.42nm). For a typical bacterial promoter, RNAP binding occurred reversibly. The mean time between RNAP binding events was measured and related to a kinetic model. The mean time between binding events defines a promoter-on rate. Once the DNA torque reaches the constant-torque regime, the promoter-on rate becomes constant.

Ideally, our model would replicate these results, with negative supercoiling promoting RNAP binding and negative supercoiling inhibiting RNAP binding, but would only depend on the local supercoiling density. Successful phenomenological models such as El Houdaigui et al. (2019) often use sigmoidal curves that are most sensitive in the range from $\sigma = -0.1$ to $\sigma = 0.1$; ideally, our model would be sensitive in a similar regime.

Dual linking number constraint model

To model the unwinding induced by RNAP binding, we add two new linking number constraints, spaced 13 base pairs apart, and analyze the energy required to perform this unwinding. To start, all of these have specific linear locations z and rotations ϕ; we will relax this restriction later.

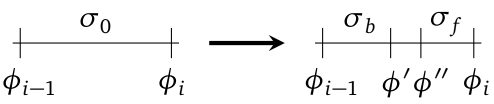

Figure S8: Diagram of the addition of two linking number constraints

We then put an angular restriction on the two new linking number constraints. By subtracting off the excess angle ϕ that the undisturbed DNA has prior to RNAP binding, we can define:

$$\Delta\phi' = \phi' - \left(\phi_{i-1} + \frac{z' - z_{i-1}}{z_i - z_{i-1}}(\phi_i - \phi_{i-1})\right) \tag{S6}$$

$$\Delta\phi'' = \phi'' - \left(\phi_{i-1} + \frac{z'' - z_{i-1}}{z_i - z_{i-1}}(\phi_i - \phi_{i-1})\right) \tag{S7}$$

For unwinding of the region bound to the RNAP to occur, we impose two constraints that ensure that the 13 base pairs that interact with the RNAP are fully unwound:

$$|\Delta\phi'| + |\Delta\phi''| = 1.2 \cdot 2\pi \tag{S8}$$

$$\Delta\phi' - \Delta\phi'' = 1.2 \cdot 2\pi \tag{S9}$$

For the intermediate region, this implies that there are two different ways to unwind the DNA; either to rotate the leading linking number constraint backwards or rotate the trailing linking number constraint forwards. There are also many intermediate solutions where *both* of the bounding linking number constraints move. The first restriction is there to ensure that we use the solution that minimizes total amount of rotation needed.

Given a complete energy model that related τ over the complete range of unwinding $\Delta\phi', \Delta\phi''$ values, we could directly write the binding energy as

$$\Delta E = \overline{\tau}'\Delta\phi' + \overline{\tau}''\Delta\phi'' \tag{S10}$$

and minimize the energy cost with respect to $\Delta\phi'$ or $\Delta\phi''$, giving a unique solution for any specific geometry. As Marko's statistical mechanical model is likely not valid in the limit of complete unwinding, we instead move forward with this model by estimating the energy cost in both the "exterior" region and within the "interior", 13-bp region.

Energy estimation in the exterior region

If we assume that the energy surface is locally linear (e.g. in the exterior region, there is a small change in supercoiling density), then we can relate the instantaneous torque to the change in energy:

$$\tau = \frac{1}{\omega_0}\frac{\partial S(\sigma)}{\partial \sigma}$$

for S, the energy per unit length. This means that:

$$\Delta E = (z_2 - z_1)\tau\omega_0\Delta\sigma = (z_2 - z_1)\frac{\partial S}{\partial \sigma}\Delta\sigma \tag{S11}$$

By expanding the definition of $\Delta\sigma$ for an arbitrary region bounded by two linking number constraints, we can actually recover the normal energy-torque definition:

$$\Delta E_{\text{external}} = (z_2 - z_1)\tau\omega_0\frac{\Delta\phi_2 - \Delta\phi_1}{\omega_0(z_2 - z_1)} = \tau(\Delta\phi_2 - \Delta\phi_1) \tag{S12}$$

Note that this implies that the energy cost of linking number constraint rotation is independent of the region width; under a locally linear assumption, the energy cost depends only on the local instantaneous torque and the rotation angle. When is this valid? The change in supercoiling density in the exterior region is relatively low, $O(0.05)$ as long as the region of interest is at least $250bp$ away from the nearest linking number constraint on one of its sides. For promoter regions analyzed here, space between gene bodies ensures that this assumption holds.

Energy estimation in the interior region (local melting)

We can generally write the energy it takes to unwind the DNA in the interior region as an integral over the linear free energy density, S:

$$\Delta E_{\text{internal}} = \int_{\sigma_0}^{\sigma_0 + \Delta\sigma_{\text{RNAP}}} \Delta z_{\text{RNAP}}\frac{\partial S}{\partial \sigma}d\sigma$$

Given the well-defined structure of the RNAP-DNA complex and the fact that under all realistic physiological conditions $\Delta\sigma_{\text{RNAP}} \gg \sigma_0$, we assume that the end-state energy is a constant, but unknown S_{unwound}:

$$\Delta E_{\text{internal}} = \Delta z_{\text{RNAP}} \left(S_{\text{unwound}} - S(\sigma_0)\right) \tag{S13}$$

While S_{unwound} is unknown, it is a constant with respect to all promoters, so this energetic term is implicitly already included in any promoter base rate. This means that the external energy depends only on the free energy density, evaluated at the local, initial supercoiling density σ_0:

$$\Delta E_{\text{internal}} = -\Delta z_{\text{RNAP}} S(\sigma_0) \tag{S14}$$

Order of magnitude energy analysis

Combining the results of eqs. (S12) and (S14) and plugging in eq. (S9), we have an estimated binding energy:

$$\Delta E = \tau(\sigma_0) 1.2 \cdot 2\pi - \Delta z_{\text{RNAP}} S(\sigma_0) \tag{S15}$$

When we plot this energetic term in fig. S6b, we see that the internal energetic term is minimal when compared to the external energetic term (local melting) at the supercoiling densities considered here, so we use the simplified form

$$\Delta E = \tau(\sigma_0) \cdot 1.2 \cdot 2\pi \tag{S16}$$

as presented in eq. (6).

Implementing the zebrafish clock gene network

In order to implement the zebrafish clock gene network, we used the stochastic reaction network and rate constants presented by Zinani et al. (2021). In brief, we add additional stochastic protein-production and protein-degradation reactions that correspond to the production of *her1* and *her7*. The protein production reactions are first-order with respect to the corresponding mRNA concentrations and the protein degradation reactions are first-order with respect to the protein concentration, and take the form:

$$r_{\textit{her1}\text{ production}} = k_{\text{protein production}} \cdot [\textit{her1}\text{ mRNA}] \tag{S17}$$
$$r_{\textit{her7}\text{ production}} = k_{\text{protein production}} \cdot [\textit{her7}\text{ mRNA}] \tag{S18}$$
$$r_{\textit{her1}\text{ degradation}} = k_{\text{protein degradation}} \cdot [\textit{her1}] \tag{S19}$$
$$r_{\textit{her7}\text{ degradation}} = k_{\text{protein degradation}} \cdot [\textit{her7}] \tag{S20}$$

As in Zinani et al. (2021), we assume the starting amount of the cofactor *hes6* is 100 proteins.

The *her1* and *her7* promoters can then be in a bound or unbound state by either the *her1*-*her1* homodimer or the *hes6*-*her7* heterodimer. Zinani et al. (2021) use a single combined dimerization and binding equation, with rates:

$$r_{\text{promoter binding (11)}} = k_{\text{dimer association}}[\text{unbound promoter}]\frac{[\text{unbound }\textit{her1}]([\text{unbound }\textit{her1}]-1)}{2} \tag{S21}$$
$$r_{\text{promoter unbinding (11)}} = k_{\text{dimer disassociation}}[\text{bound promoter}_{11}] \tag{S22}$$
$$r_{\text{promoter binding (67)}} = k_{\text{dimer association}}[\text{unbound promoter}][\text{unbound }\textit{hes6}][\text{unbound }\textit{hes7}] \tag{S23}$$
$$r_{\text{promoter unbinding (67)}} = k_{\text{dimer disassociation}}[\text{bound promoter}_{67}] \tag{S24}$$

Note that the bound/unbound promoter species variables take on values zero or one, for each promoter. For example, if the *her1* promoter is currently bound by a *her1*-*her1* homodimer, then the unbound variable [unbound *her1* promoter] $= 0$, but the bound variables take values [bound $\textit{her1}_{11}$] $= 1$, [bound $\textit{her1}_{67}$] $= 0$.
